# A review of Bornean Micronectidae (Hemiptera, Heteroptera, Nepomorpha) with descriptions of two new species from Sabah, Malaysia^1^

**DOI:** 10.3897/zookeys.501.9416

**Published:** 2015-04-30

**Authors:** Ping-ping Chen, Nico Nieser, Johnny Lapidin

**Affiliations:** 1 Naturalis Biodiversity Centre, P.O. Box 9517, 2300 RA Leiden, The Netherlands; 2 Division of Research and Education, Headquarter of Sabah Parks, 88806 Kota Kinabalu, Sabah, Malaysia

**Keywords:** Hemiptera, Micronectidae, *Micronecta*, new species, key, Borneo

## Abstract

Previous research of Bornean Micronectidae Jaczewski, 1924 (pygmy water boatmen) is summarized based on the data from the literature and recent work. All the Bornean micronectids belong to the genus *Micronecta* Kirkaldy, 1897. Descriptions or redescriptions and a key to the eight species, which have so far been found in Borneo are presented, namely *Micronecta decorata* Lundblad, 1933, *Micronecta ludibunda* Breddin, 1905, *Micronecta liewi* **sp. n.**, *Micronecta lakimi* **sp. n.**, *Micronecta lumutensis* Chen, Nieser & Lansbury, 2008, *Micronecta skutalis* Nieser & Chen, 1999, *Micronecta kymatista* Nieser & Chen, 1999) and *Micronecta quadristrigata* Breddin, 1905. The synonyms are indicated under each species. To facilitate identification, illustrations and habitus photos are provided. The faunistic components of Micronectidae in Borneo are discussed from a zoogeographic point of view.

## Introduction

**Figures 1–2.**
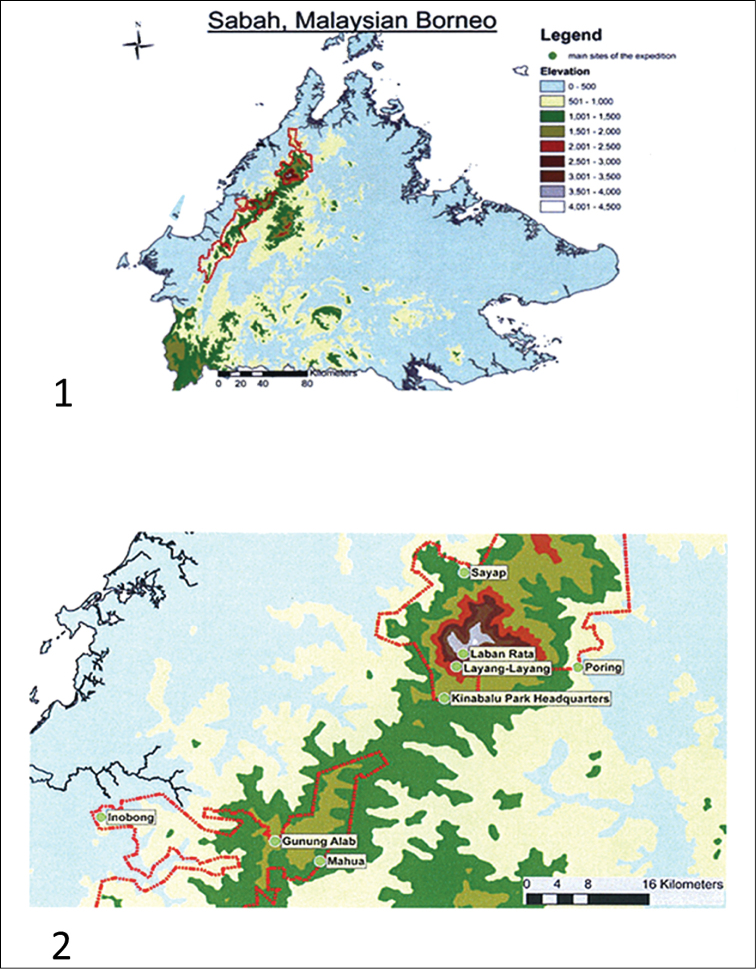
**1** Map of Sabah, (from Kitaura et al. 2003), indicating the excursion area in the Sabah Parks in 2012 **2** The substations in Crocker Range and Mt. Kinabalu National Parks (from Kitaura et al. 2003), with indications the localities of the samples. Credit: Sabah Parks.

**Figures 3–4.**
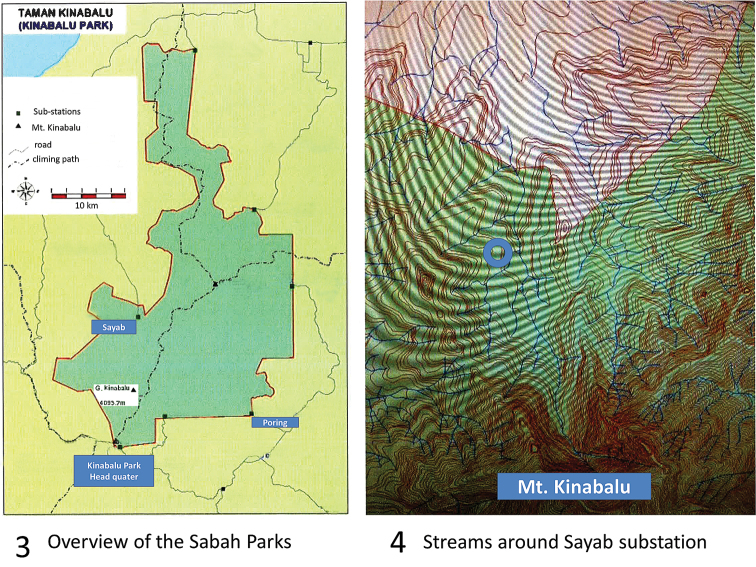
**3** The area of Sabah Parks, with indications of the sampling area **4** The rivers and streams around the Sayab Substation, with the indication of the sampled sites. Credit: Sabah Parks.

The Scientific Expedition to Mount Kinabalu–Crocker Range in September 2012 (http://kinabalu-expedition.blogspot.nl/), organized jointly by Sabah Parks, Malaysia and the Naturalis Biodiversity Centre (NBC), The Netherlands, offered us an opportunity to collect water bugs at several substations in the Parks. The result has led to a better understanding of the water bug fauna in the area, including the discovery of several undescribed species. As a result of the expedition, a review of the Sabah Micronectidae is presented. For locations and the ground plan of the Sabah Parks, see Figs 1–4.

**Figures 5–10.**
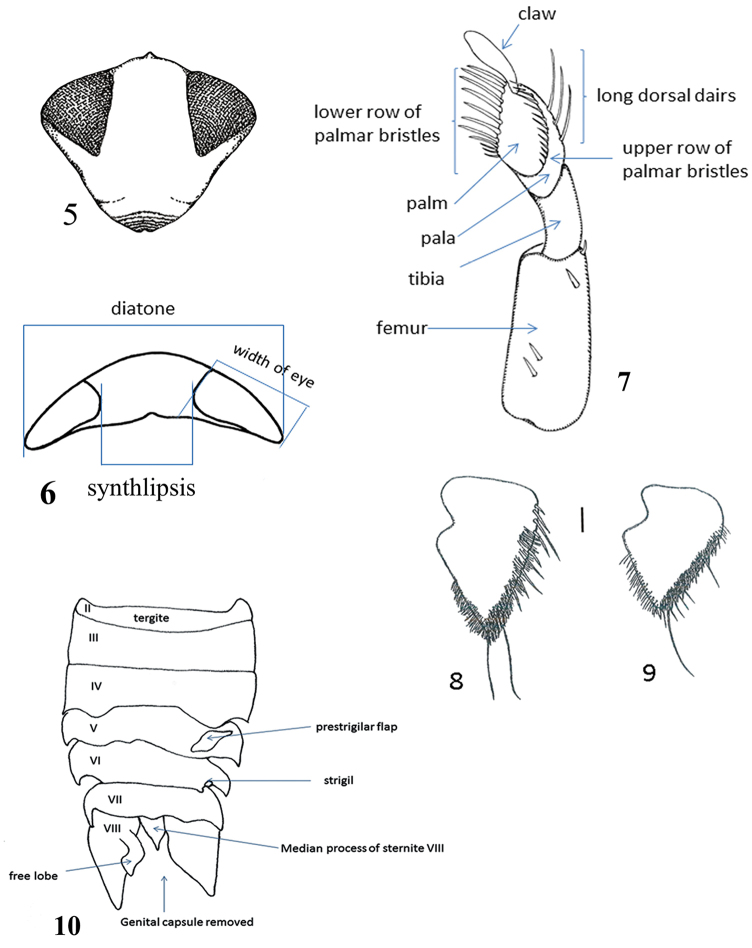
**5–7, 10** *Micronecta* sp. diagrammatical illustrations of morphological terms used in the text: **5** head in frontal view **6** head in dorsal view **7** fore leg **8–9** *Micronecta* spp. right part of tergite VIII of males, in dorsal view, scale 0.1 mm: **8** *Micronecta kymatista* Nieser & Chen, 1999 **9** *Micronecta quadristrigata* Breddin, 1905 **10** *Micronecta* sp. schematic dorsal view of male abdominal segments.

**Figure 11.**
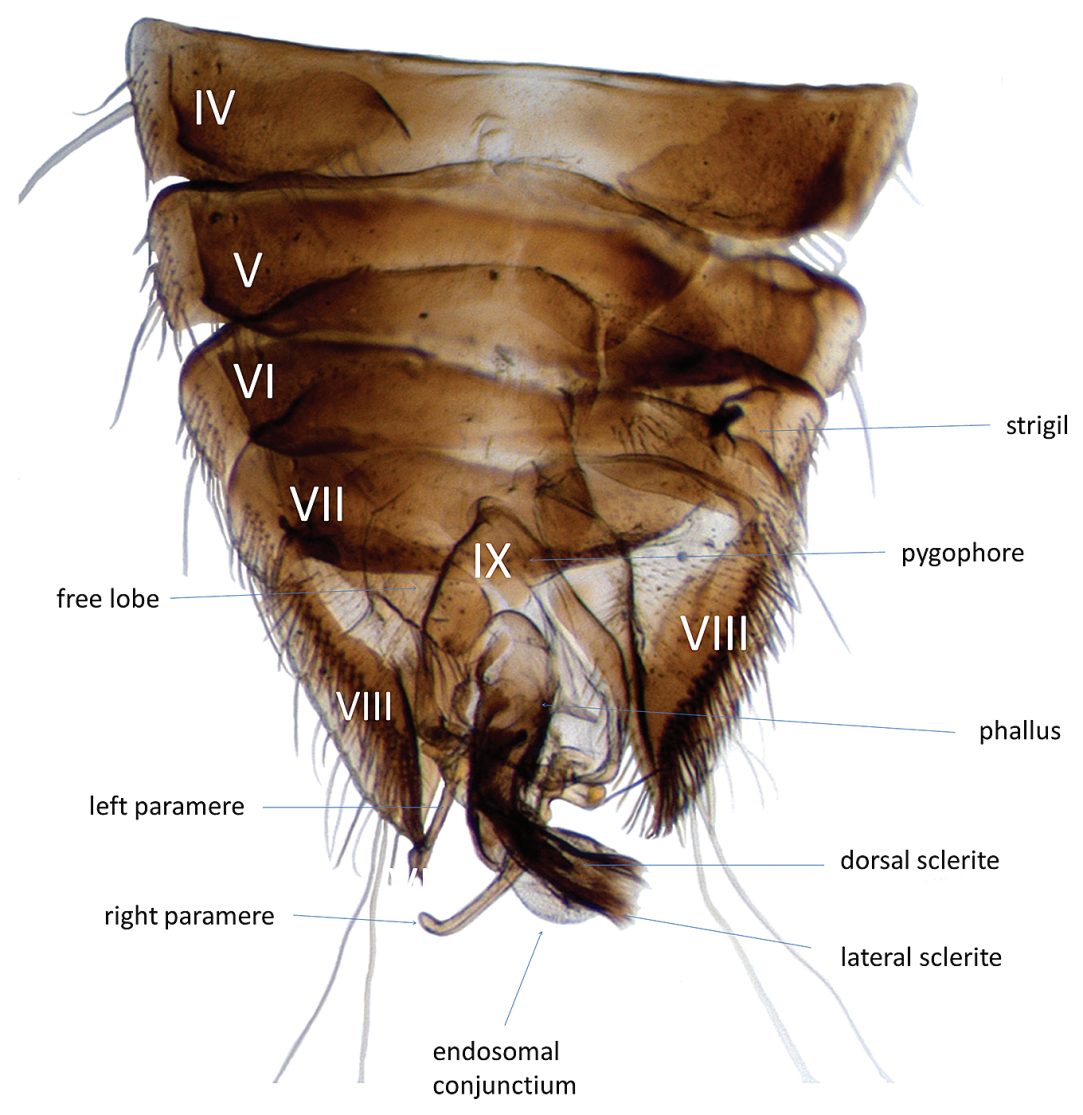
*Micronecta* sp., male, abdomen in dorsal view.

**Figures 12–13.**
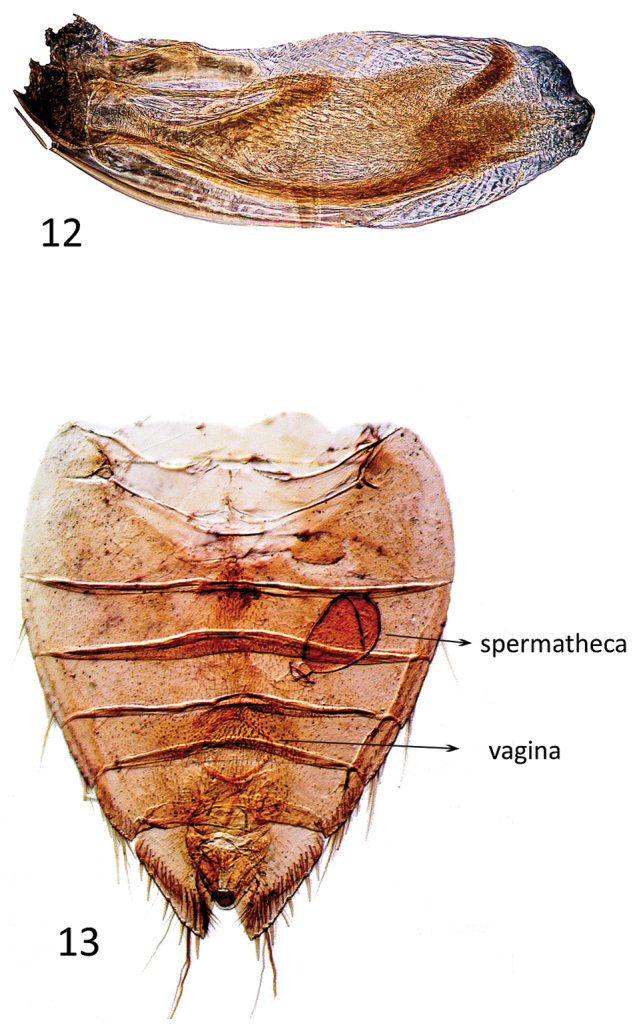
**12** *Micronecta* sp., male phallus **13** *Micronecta* sp. female abdominal segments in ventral view, indicating the genitalia structures.

The Micronectidae (pygmy water boatmen) belong to the superfamily Corixoidea (Leach, 1815), which is in the infraorder Nepomorpha Popov, 1971. Most species in the Nepomorpha live in water and are characterized by the antennae implanted under the head. In the most obligate aquatic species, their antennae are shorter than the head and not visible in dorsal view. Within Nepomorpha, the Corixoidea are recognized by the broadly triangular, unsegmented rostrum, although transverse grooves are present in most species (Fig. 5). The abdominal structure in males is strongly modified in Micronectidae as in other Corixoidea taxa, with segments V–VIII asymmetrical (Figs 10, 11). The male genitalic structures (Figs 11, 12) are similar to those of *Sigara* Fabricius, 1775, in the Corixidae: Corixinae. The females have a unique spermatheca (Fig. 13) by having a large distal seminal receptacle among water bugs (Larsen 1938, and Pluot-Sigwalt, personal communication).

Micronectids are small bugs with a body length less than 5 mm. The Bornean species are all less than 3.5 mm long. Most species of Micronectidae occur in the tropics and subtropics, with only a few found in temperate or cold climates of the Palaearctic Region. The Micronectidae can be easily separated from Corixidae (Leach, 1915) by the following characteristics: scutellum exposed, not covered by the pronotum, and the absence of ocelli. Micronectids are usually found in shallow stagnant or virtually stagnant habitats. Most species seem to prefer an open sandy or clayey bottom with little or no plant debris. In our experience, they can be especially numerous in shallow edges of ponds with sandy bottoms in temperate regions, and in open shallow pools of stream beds with sandy bottoms in tropical areas (Figs 98, 99).

## The history of Bornean Micronectidae

Although the history of studying of micronectids can be traced back to Linnaeus more than 200 years ago, the Bornean fauna of Micronectidae remains poorly known. Wróblewski (1968) speculated that *Micronecta decorata* Lundblad, 1933 might be present in Borneo. Only the recent expeditions to Borneo by NCB Naturalis have led to the first confirm records of micronectids on the island. Three species were found in 1999 (*Micronecta ludibunda* Breddin, 1905; *Micronecta kymatista* Nieser & Chen, 1999; *Micronecta skutalis* Nieser & Chen, 1999); and later *Micronecta lumutensis* Chen et al., 2008 was described from Kalimantan. The last expedition in 2012 exploring the mountainous areas of the Sabah Parks added two species new to science, one species new to Borneo, and the confirmation of *Micronecta decorata* on the island.

## Material and methods

Mount Kinabalu gives rise to five catchments (Wong and Philipps 1996). Sungai Silau–Silau and its tributary, the small stream of Carson Falls, originate in the Headquarters (Figs 2–3) area and flow into the Sungai Liwagu, which originates on the south slope near Headquarters and discharges into the Labuk River, which flows eastward into Labuk Bay north of Sandakan. Likewise, the Sungai Kipungit at Poring (Figs 2–3) ultimately discharges into Sungai Labuk. Our samples CN1268, CN1270, CN1271, CN1272 and CN1274 are from this catchment. The Sungai Kadamaian originates up-mountain from Kampong Kiau, and its tributary Sungai Kematis up-mountain from Kampong Sayap (Figs 2–4). The Sungai Kadamaian flows northwestward past Kota Belud into the South China Sea. Samples CN1262, CN1263, CN1264, and CN1275 are from the Sungai Kadamaian catchment area. Finally Sungai Kibambang and Sungai Mahua, which originate in the Crocker Range (Fig. 2) flows into Sungai Pegalan which joins Sungai Padas before draining into Brunei Bay. Samples CN1277, CN1278, CN1279, CN1281, CN1283, CN1285, CN1286, CN1288, and CN1289 are from the Sungai Pegalan catchment area.

The specimens obtained in Sabah were collected with a hand net, unless otherwise indicated in the material examined sections. The number of net sweeping or the time spent one locality was not standardized. We usually collected in a given locality until three subsequent netting hauls did not yield any additional species. When unusual specimens were collected, an additional effort was made to collect a longer series. Most studied specimens are preserved in 96% ethanol, but some were mounted on carton labels or on microscopic slides.

To facilitate working with the key and better understanding the descriptions, three diagrammatic figures (Figs 6, 7, 10) and a photograph (Fig. 11) of the male genitalic structures of *Micronecta* sp. are provided. Anatomical abbreviations and terms used in species descriptions are indicated in Figs 5–13.

Specimens were studied by using a binocular (Zeiss Stemi 2000) and a compound microscope (Olympus BX51). Measurements are in mm, based on five specimens of each sex from the series (including the holotype, if available) and presented as a size range. Ocular index is 2S/ (D–S). Photographs were taken with Zeiss Discovery V12 SteRIO, lens Zeiss Plan Apo S 1.0×, FWD 60 mm, and, if necessary, were further processed using Adobe Photoshop CS6. Line illustrations were made using a binocular Zeiss Stemi 2000 with a camera lucida.

The studied specimens from several museum collections were mainly caught at light. The holotypes of the newly described species are placed in the Naturalis Biodiversity Centre (RMNH); the remaining material collected in Sabah is divided over NCTN, NMPC, RMNH, and ZCSM.

The following acronyms of museum collections are used:

NCTN Nieser & Chen Collection, Tiel, The Netherlands;

NHMW Naturhistorisches Museum Wien, Vienna, Austria;

NMPC National Museum (Natural History), Praha, Czech Republic;

RMNH Naturalis Biodiversity Centre, Leiden, The Netherlands;

ZCSM Zoological Collection of The Sabah Parks, Sabah, Malaysia;

ZMHB Museum für Naturkunde der Humboldt Universität zu Berlin, Bereich Zoologisches Museum, Berlin, Germany;

ZMUH Zoologisches Institut und Zoologisches Museum, Universität Hamburg, Hamburg, Germany.

### Key to species of *Micronecta* occurring in Borneo (mainly applicable to males)

**Table:** 

1	Corium with four solid longitudinal, darker stripes with variation from weak to distinct rings; pronotum typically with a pair of oval rings, varying from virtually absent to distinct (Fig. 12); left paramere with a laterally compressed tip (Fig. 77). Body length 1.9–2.4 mm	**Micronecta (Dichaetonecta) ludibunda Lundblad, 1933**
–	Corium with or without broken longitudinal stripes; pronotum without darker markings	**2**
	(Remarks: Some specimens of *Micronecta kymatista* and *Micronecta quadristrigata* may have fairly distinct longitudinal stripes on the corium, but these species have the left paramere with a sickle-shaped apex (Figs 87, 89); and are larger on average with lengths of 2.2–3.1 mm)	
2	Smaller species, body length less than 2.0 mm	**3**
–	Larger species, body length 2.0 mm or more	**5**
3	Hemelytra with a broad transverse medium to dark brown band at middle (Figs 17, 26); left paramere with a ribbed apex (Fig. 83). Body length 1.7–1.8 mm	**Micronecta (Micronecta) liewi sp. n.**
–	Hemelytra without a broad transverse medium to dark brown band; left paramere not ribbed apically. Body length 1.5–1.7 mm	**4**
4	Left paramere with a rounded apex and a small indentation at the base of the shaft (Fig. 85). Body length 1.5–1.7 mm	**Micronecta (Micronecta) skutalis Nieser & Chen, 1999**
–	Left paramere with an indented apex and without a small indentation at the base of the shaft (Fig. 79). Body length 1.5 mm	**Micronecta (Micronecta) lumutensis Chen, Nieser & Lansbury, 2008**
5	Free lobe of tergite VIII straight, with a sinuate apical margin (Fig. 70); apex of left paramere not sickle-shaped (Fig. 81)	**6**
–	Free lobe of tergite VIII sinuate with a rounded apical margin (Fig. 72); apex of left paramere sickle-shaped (Fig. 87)	**7**
6	Species dark brown; free lobe of tergite VIII apically narrowed (Fig. 70); right paramere apically dilated (Fig. 80). Body length 2.0–2.2 mm	**Micronecta (Micronecta) lakimi sp. n.**
–	Species light to medium brown; free lobe of tergite VIII apically widened (Fig. 66); apex of right paramere acutely narrowed (Fig. 74). Body length 2.2–2.4 mm	**Micronecta (Dichaetonecta) decorata Lundblad, 1933**
7	Apical half of inner margin of right part of tergite VIII with 28–35 marginal hairs caudally arranged in a double or triple row (Fig. 8). Body length 2.8–3.1 mm	**Micronecta (Sigmonecta) kymatista Nieser & Chen, 1999**
–	Apical half of inner margin of right part of tergite VIII with 20–25 marginal hairs caudally arranged in a single to double row (Fig. 9). Body length 2.2–2.9 mm	**Micronecta (Sigmonecta) quadristrigata Breddin, 1905**

## Descriptions and redescriptions of the species of *Micronecta* in Borneo

### Genus Micronecta

Taxon classificationAnimaliaHemipteraMicronectidae

Kirkaldy, 1897

#### Type species.

*Notonecta minutissima* (Linnaeus, 1758), by original designation.

### Subgenus Dichaetonecta

Taxon classificationAnimaliaHemipteraMicronectidae

Hutchinson, 1940

#### Type species.

*Sigara scholtzi* Fieber, 1860, by original designation.

#### Diagnosis.

Male: palar claw usually of moderate size, strigil present, seventh abdominal sternite with one or two strongly developed bristles, prestrigilar flap with a very obtuse tip, left paramere variable but not with a plate-like shaft with sub parallel margins, right paramere elongate.

### Micronecta (Dichaetonecta) decorata

Taxon classificationAnimaliaHemipteraMicronectidae

Lundblad, 1933

Figs 14 , 24 , 30 , 31 , 43 , 51 , 58 , 66 , 74 , 75 , 90

Micronecta decorata Lundblad, 1933: 93–94 (original description).
Micronecta decorata : Wróblewski 1968: 775 (checklist).
Micronecta decorata : Nieser 2000: 287 (key).
Micronecta decorata : Chen et al. 2005: 420 (checklist).

#### Material examined.

**THAILAND (new record for Thailand): Chiang Mai Province:** Doi Saket, Ban Pong Ao, Kuang River at bridge in road 118, 38 km NE Chiang Mai City, 30.i.2002, leg. P. Chen, N. Nieser, A. Thanyakam & C. Duangsupa, C0220, 19 males 30 females. **Uttaradit Province:** Baan Muangchedton, Lake Naam Pat, 10 km W of Ban Khok town, 10.ii.2002, stagnant ponds downstream of barrage, 10.ii.2002, leg. P. Chen, N. Nieser, A. Thanyakam, C. Duangsupa & W. Jaiyai, C0231, 7 males 13 females. All macropterous (samples stored in ethanol 70%). **MALAYSIA: Sabah (confirmation of occurrence in Borneo):** Kota Belud Dist., Crocker Range Park, Sungai Mahua at substation beside restaurant, 05°47.53'N, 116°24.19'E, 1053 m. a.s.l., 22.ix.2012, leg. P. Chen, N. Nieser & J. Lapidin, CN1283, 1 male and 1 female macropterous. (All are in the collection of NCTN).

#### Redescription.

**Figures 14–21.**
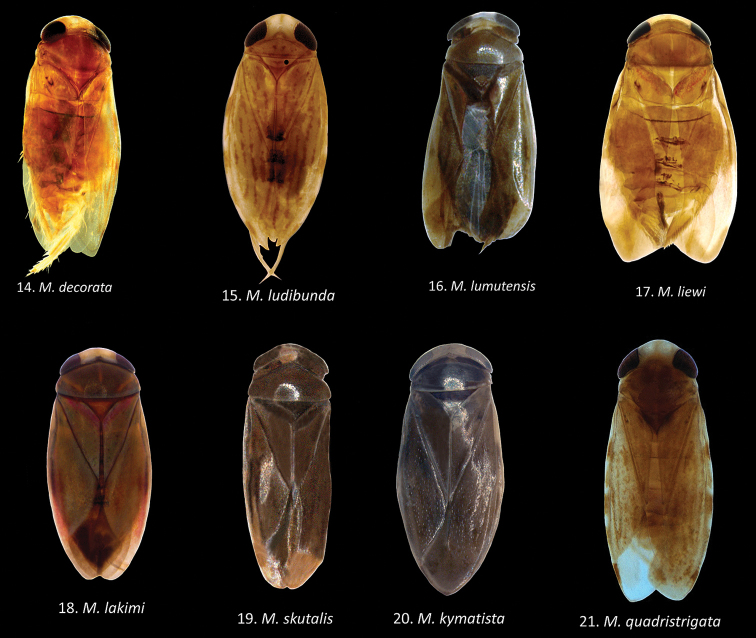
Habitus of *Micronecta* spp., in dorsal view, legs omitted: **14** *Micronecta decorata* Lundblad, 1933, macropterous male, body length 2.38 mm **15** *Micronecta ludibunda* Breddin, 1905, brachypterous male, body length 2.32 mm **16** *Micronecta lumutensis* Chen, Nieser & Lansbury, 2008, paratype, macropterous male, body length 1.40 mm **17** *Micronecta liewi* sp. n., paratype, macropterous male, body length 1.77 mm **18** Micronecta (Micronecta) lakimi sp. n., paratype, macropterous male, body length 2.12 mm **19** *Micronecta skutalis* Nieser & Chen, 1999, paratype, macropterous male (membrane rolled partly inward), body length 1.58 mm **20** *Micronecta kymatista* Nieser & Chen, 1999, paratype, macropterous male, body length 2.80 mm **21** *Micronecta quadristrigata* Breddin, 1905, macropterous male, body length 2.88 mm.

Macropterous specimens. Generally a medium-sized, (length 2.2–2.4 mm) yellowish-brown species, with darker markings varying from virtually absent (Fig. 14) to quite distinct, medium- brown: a V-shaped stripe on clavus and four interrupted longitudinal stripes on corium (Fig. 24); eyes castaneous to grayish.

*Dimensions*. Body length: male 2.2–2.3, female 2.2–2.4; width: male 1.01–1.06, female 1.00–1.18; diatone: male 0.77–0.81, female 0.75–0.84; width of pronotum: male 0.82–0.88, female 0.81–0.93; ocular index: male 1.56–1.77, female 1.48–1.65. Body length twice the maximal width (male 2.23/1.04, female 2.33/1.12). Pronotum slightly wider than head (H/P male 0.80/0.85, female 0.81/0.88), synthlipsis one and half times the posterior width of an eye (S/E male 0.37/0.21, female 0.36/0.24).

*Colour*. Frons and vertex sordid yellow, eyes castaneous to grayish. Pronotum yellowish-brown, disk without markings except for a distinct yellowish stripe on posterior margin. Hemelytra light brown, with elongate darker marks arranged in four interrupted, longitudinal, brown stripes on corium (Fig. 24). Right membrane slightly paler than corium, without markings; left membrane hyaline. Embolium yellowish brown with three brown spots. Venter, abdomen, thorax, and legs pale yellow. [Our Thai material contains specimens with only a very vague or virtually absent hemelytral pattern. The Borneo specimens show a hemelytral pattern similar to *Micronecta quadristrigata* as stated by Lundblad (1933). Apparently, the hemelytral pattern fades when the specimens are stored in 70% or 96% ethanol].

*Pronotum*. About two and a half times as wide as long (W/L 0.87/0.36), dorsally convex with lateral margins straight and more or less truncate (Fig. 14). Hemelytra smooth, with four shallow, longitudinal grooves on corium, densely beset with small spinules, notably on corium. The right membrane texture same as corium, smooth without grooves or spines. Spines laterally on abdominal segments: V with two short and one longer stout spine; VI with three short and one long spine; VII with two or three short and one long stout spine; VIII with four or five short and one long, stout spine or sometimes without a long spine and two long hair-like bristles.

*Legs*. Length of segments: fore leg: male: femur 0.28, tibia 0.14, pala 0.15; female: femur 0.31, tibiotarsus 0.30; middle leg: male: femur 0.85, tibia 0.27, tarsus 0.39, claw 0.30; female: femur 0.85, tibia 0.29, tarsus 0.40, claw 0.28; hind leg: male: femur 0.51, tibia 0.40, tarsus I 0.39, tarsus II 0.19, claw 0.13; female: femur 0.52, tibia 0.42, tarsus I 0.37, tarsus II 0.19, claw 0.12. Palmar bristles: 21–23 in upper row, 17–18 in lower row.

*Male*. Fore femur (Fig. 30), with a pair of pegs on proximal third, a small peg distally, and a larger bristle-like spine sub-distally; pala with four long dorsal hairs. Claw (Fig. 31) parallel sided, with a transverse carina. Dorsum of abdomen: prestrigilar lobe sub-triangular, with a short, obtusely rounded apex (Fig. 43); strigil small, sub-oval, comb with about 45 comparatively distinct teeth (Fig. 51); free lobe of left part of tergite VIII with an expanded apex (Fig. 66), a sinuate apical margin, and 10–15 apical bristles. Left paramere (Fig. 75) with a narrow, apically, slightly dilated shaft and a subapical indentation; right paramere in lateral view (Fig. 74) with an evenly curved, sickle-shaped shaft, apex acutely tapering, basal lobe with about 25 stridulatory ridges. Mediocaudal lobe of sternite VII (Fig. 58) with apical part acutely pointed, with one strongly developed bristle.

*Female*. Fore femur with the same general arrangement of pegs and setae as in male. The seminal capsule of spermatheca clavate (Fig. 90).

#### Comparative notes.

Males can be recognized by the form of the free lobe of tergite VIII. The palmar claw of the male, with its oblique carina, also is unique but it is often folded into the palm, usually making it difficult to observe.

#### Habitat.

We have taken this species several times in Chiang Mai and other northern provinces in Thailand, where it is apparently quite common. Sample C0220 was taken from shallow virtually stagnant water in a wide unshaded river bed with a sandy bottom.

#### Distribution.

Thailand (see above); Malay Peninsula (Wróblewski 1968: record for Malaysia without exact locality; Fernando and Cheng 1974); INDONESIA: Sumatra (Lundblad 1933), Java (Lundblad 1933); and Borneo.

#### Note.

Wróblewski (1968) recorded this species from Borneo with a question mark. His speculation is confirmed here.

### Micronecta (Dichaetonecta) ludibunda

Taxon classificationAnimaliaHemipteraMicronectidae

Breddin, 1905

Figs 15 , 22 , 25 , 32 , 33 , 44 , 52 , 59 , 67 , 76 , 77 , 91

Micronecta ludibunda Breddin, 1905a: 57 (original description).
Micronecta ludibunda : Breddin 1905b: 157–158 (extensive description).
Micronecta graphiptera Horváth, 1918: 146 (original description).
Micronecta ludibunda : Lundblad 1933: 95–96 (redescription).
Micronecta inconspicua Lundblad, 1933: 96–98 (original description).
Micronecta striatella Lundblad, 1933: 98–100 (original description).
Micronecta ludibunda : Wróblewski 1968: 765–767 (redescription)
Micronecta ludibunda : Nieser and Chen 1999: 80 (record from Kalimantan Timur)
Micronecta ludibunda : Polhemus and Golia 2006: 531–534 (occurrence in Florida, USA).
Micronecta ludibunda : Tinerella 2008: 29–34 (redescription, extensive synonymy).

#### Type material examined.

Syntypes, INDONESIA: “Kotype; Buitenzorg (= Borgor) Java, K. Kraepelin; leg. 24.II–12.III.1904, ded.8.VI.1904; Breddin determ.; Lundblad revid. 1934”, 2 males 2 females (ZMUH).

#### Additional material examined.

**THAILAND: Chon Buri Province:** Khao Khaew Open Zoo, ponds, 7.iv.2001, leg. P. Chen, S. Leepitakrat & B. Kavinseksan, 50 males 50 females (sample stored in 70% ethanol in NCTN).

#### Redescription.

**Figures 22–23.**
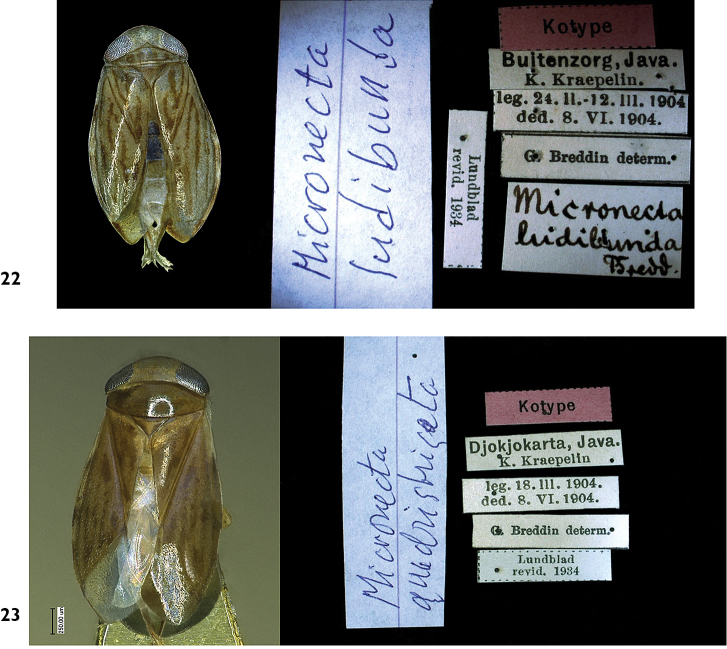
The syntypes in ZMUH, Germany: **22** *Micronecta ludibunda* Breddin, 1905, syntype, brachypterous female, body length 1.80 mm **23** *Micronecta quadristrigata* Breddin, 1905, syntype, macropterous female, body length 2.85 mm.

Brachypterous and macropterous specimens. Generally a medium-sized (length 1.9–2.4 mm), yellowish-brown, species with four distinct, uninterrupted, longitudinal stripes on corium (Figs 15, 22, 25), and a variable darker pattern on pronotum, typically consisting of a pair of oval rings. Brachypterous and macropterous specimens differ in the development of the pronotum, but the differences between the brachypterous and macropterous morph are less pronounced than in most other species of *Micronecta*.

*Dimensions*. Body length: brachypterous male 1.9–2.2, macropterous male 2.1–2.3, brachypterous female 1.9–2.3, macropterous female 2.2–2.4; width: male 1.01–1.18, female 1.04–1.22; diatone: male 0.68–0.85, female 0.70–0.87; width of pronotum: male 0.69–0.87, female 0.71–0.92; ocular index: male 1.02–1.18, female 0.87–1.14. Body length twice the maximal width (male 2.06/1.05, female 2.25/1.15). Pronotum slightly wider than head (H/P male 0.76/0.78, female 0.77/0.81), synthlipsis subequal to the posterior width of an eye (S/E male 0.24/0.24, female 0.26/0.28).

*Colour*. Frons and vertex sordid yellow, eyes castaneous. Pronotum yellowish brown, disk typically with a pair of darker oval rings, varying from nearly absent via fragmented rings to complete; posterior margin with a distinct yellowish stripe. Hemelytra yellowish brown; clavus with a darker, V-shaped, medium-brown stripe; corium typically with four longitudinal, medium-brown, uninterrupted stripes (Figs 15, 22, 25); embolium yellowish with four or five brown spots; right membrane poorly delimited from the corium, with the same colour and texture but without darker stripes; left membrane more distinctly separated from corium, hyaline, and more membranous than corium. Venter, abdomen, thorax, and legs pale yellow.

**Figures 24–29.**
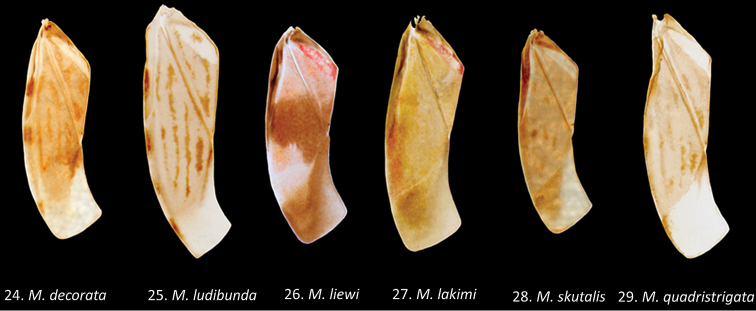
*Micronecta* spp., left forewings, in dorsal view: **24** *Micronecta decorata* Lundblad, 1933 **25** *Micronecta ludibunda* Breddin, 1905 **26** *Micronecta liewi* sp. n., paratype **27** *Micronecta lakimi* sp. n., paratype **28** *Micronecta skutalis* Nieser & Chen, 1999 **29** *Micronecta quadristrigata* Breddin, 1905.

*Pronotum* short (Fig. 15), about four times as wide as long (W/L 0.79/0.20); in brachypterous specimens dorsally flat, in macropterous specimens dorsally somewhat convex. Hemelytra smooth, sparsely beset with small spinules, notably on corium. Spines laterally on abdominal segments: V with two short and one longer stout spine; VI with two short, and one long spine; VII with two or three short, and one long stout spine; VIII with five short and one long stout spine, and one long hair-like bristle.

*Legs*. Length of leg segments: fore leg: male: femur 0.26, tibia 0.14, pala 0.14; female: femur 0.26, tibiotarsus 0.26; middle leg: male: femur 0.70, tibia 0.23, tarsus 0.30, claw 0.25, female: femur 0.76, tibia 0.23, tarsus 0.33, claw 0.26; hind leg: male: femur 0.46, tibia 0.36, tarsus I 0.40, tarsus II 0.13, claw 0.08; female: femur 0.48, tibia 0.37, tarsus I 0.42, tarsus II 0.16, claw 0.08. Palmar bristles: 10 to 11 in upper row, 10 to 11 in lower row.

*Male*. Fore femur (Figs 32, 33) with a pair of pegs on proximal third and a pair of small pegs distally; pala with three long dorsal hairs. Claw slender and clavate, apex mucronate. Dorsum of abdomen: prestrigilar lobe (Fig. 44) sub-triangular, with a short, truncate apex; strigil (Fig. 52) small, suboval, comb with about 55 comparatively distinct teeth; free lobe of left part of tergite VIII (Fig. 67) with a slightly expanded apex and 10–15 apical bristles. Left paramere (Fig. 77) with a narrow, more or less parallel-sided shaft, apex laterally compressed, flag-like; right paramere in lateral view (Fig. 76) with an evenly curved shaft and tapering apex, basal lobe not distinctly differentiated from basal part of paramere, with over 50 stridulatory ridges. Mediocaudal lobe of sternite VII (Fig. 59) long, with apical part elongate and obtusely rounded to pointed apically, with or without one to two larger bristles.

**Figures 30–42.**
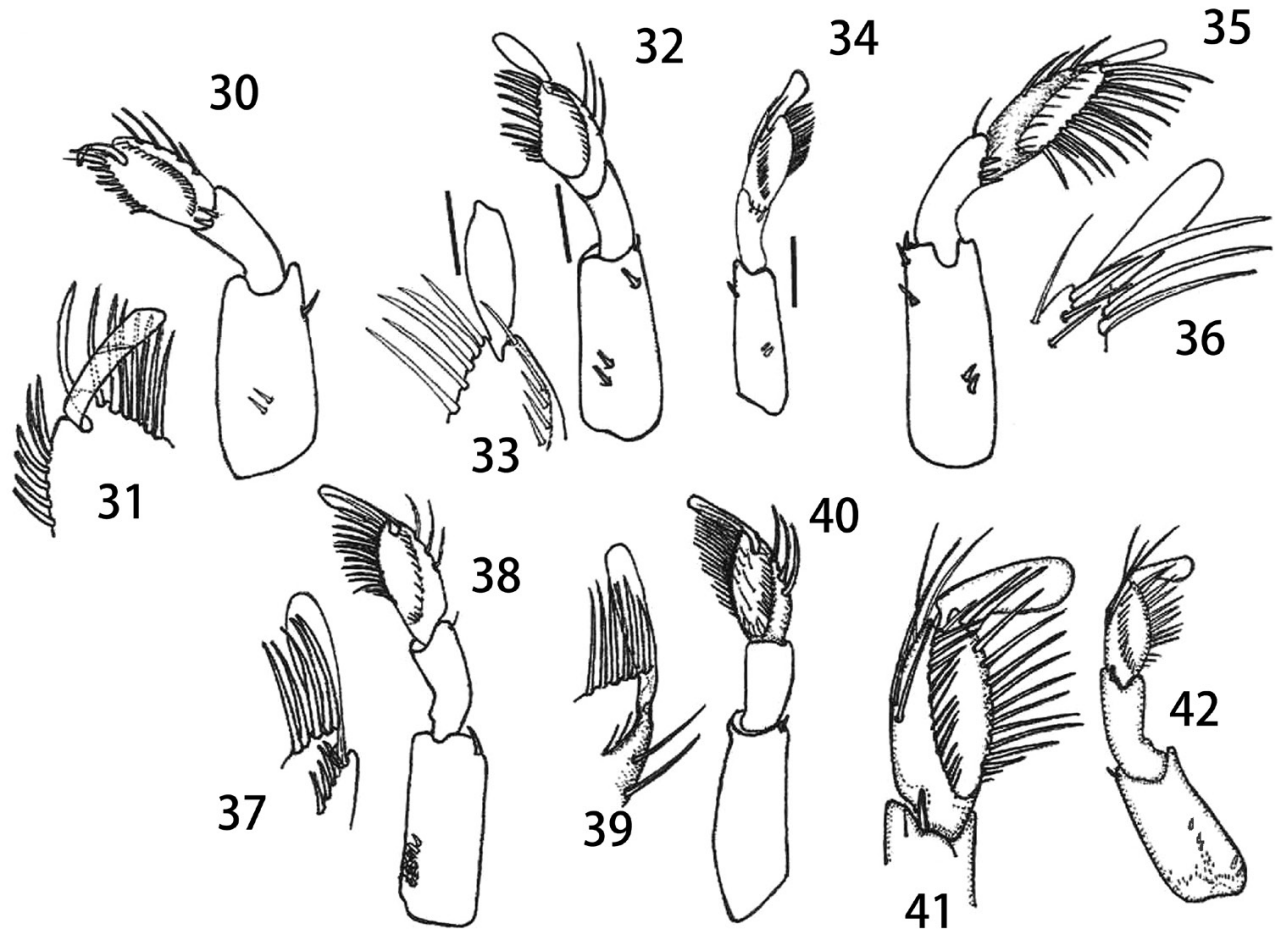
*Micronecta* spp., right foreleg in anteroventral view including apex of pala: **30–31** *Micronecta decorata* Lundblad, 1933 **32–33** *Micronecta ludibunda* Breddin, 1905 **34** *Micronecta lumutensis* Chen, Nieser & Lansbury, 2008 **35–36** *Micronecta liewi* sp. n., paratype **37–38** *Micronecta lakimi* sp. n., paratype **39–40** *Micronecta skutalis* Nieser & Chen, 1999 **41** *Micronecta kymatista* Nieser & Chen, 1999 **42** *Micronecta quadristrigata* Breddin, 1905. Scale bars: 0.1 mm (**30, 32, 34, 35, 37, 39, 42**); 0.05 mm (**31, 33, 36, 38, 40, 41**).

**Figures 43–50.**
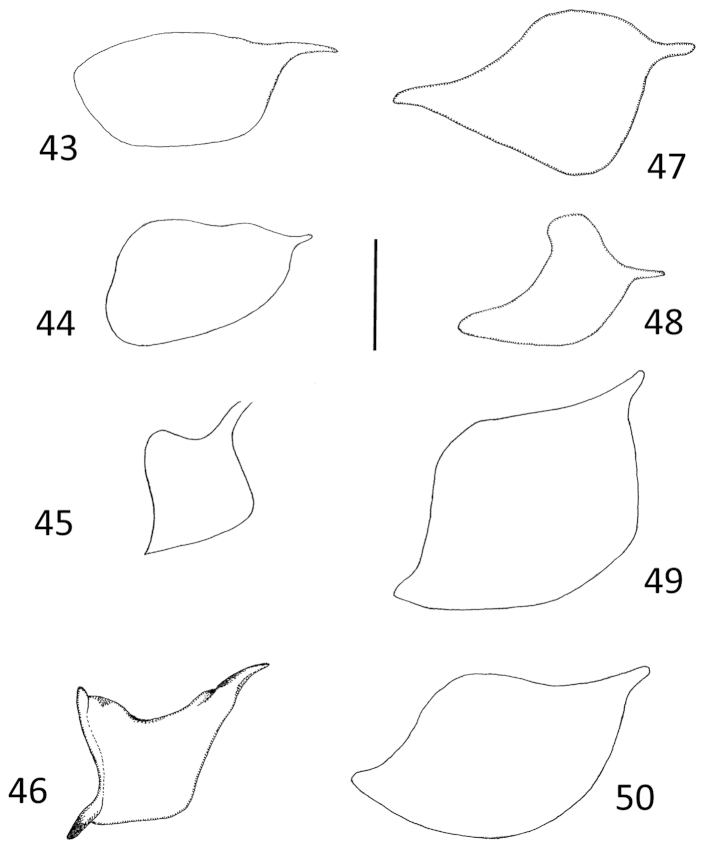
*Micronecta* spp., prestrigilar flap on abdominal segment V, male; in dorsal view: **43** *Micronecta decorata* Lundblad, 1933 **44** *Micronecta ludibunda* Breddin, 1905 **45** *Micronecta lumutensis* Chen, Nieser & Lansbury, 2008 **46** *Micronecta liewi* sp. n., paratype **47** *Micronecta lakimi* sp. n., paratype **48** *Micronecta skutalis* Nieser & Chen, 1999 **49** *Micronecta kymatista* Nieser & Chen, 1999 **50** *Micronecta quadristrigata* Breddin, 1905. Scale bar: 0.1 mm

**Figures 51–57.**
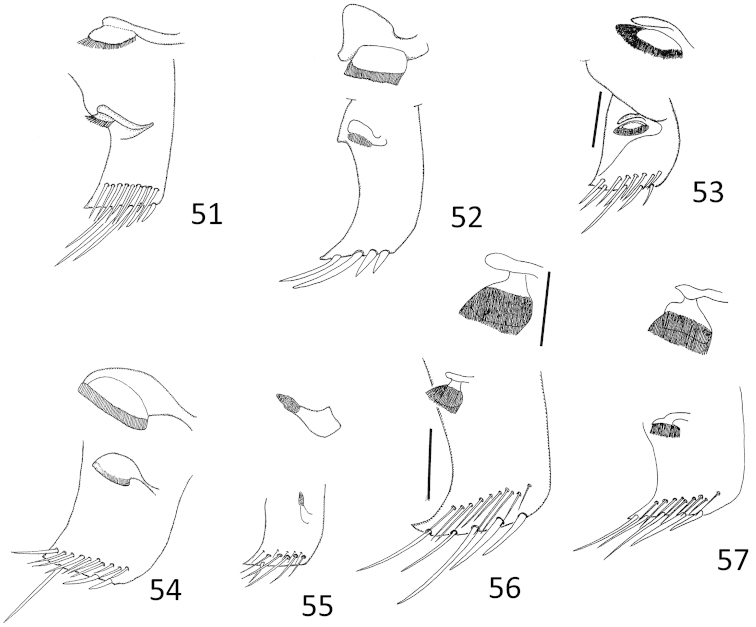
*Micronecta* spp., right part of tergite VI with strigil (scale 0.05 mm), males, in dorsal view: **51** *Micronecta decorata* Lundblad, 1933 **52** *Micronecta ludibunda* Breddin, 1905 **53** *Micronecta liewi* sp. n., paratype **54** *Micronecta lakimi* sp. n., paratype **55** *Micronecta skutalis* Nieser & Chen, 1999 **56** *Micronecta kymatista* Nieser & Chen, 1999 **57** *Micronecta quadristrigata* Breddin, 1905. Scale bars: 0.1 mm

**Figures 58–65.**
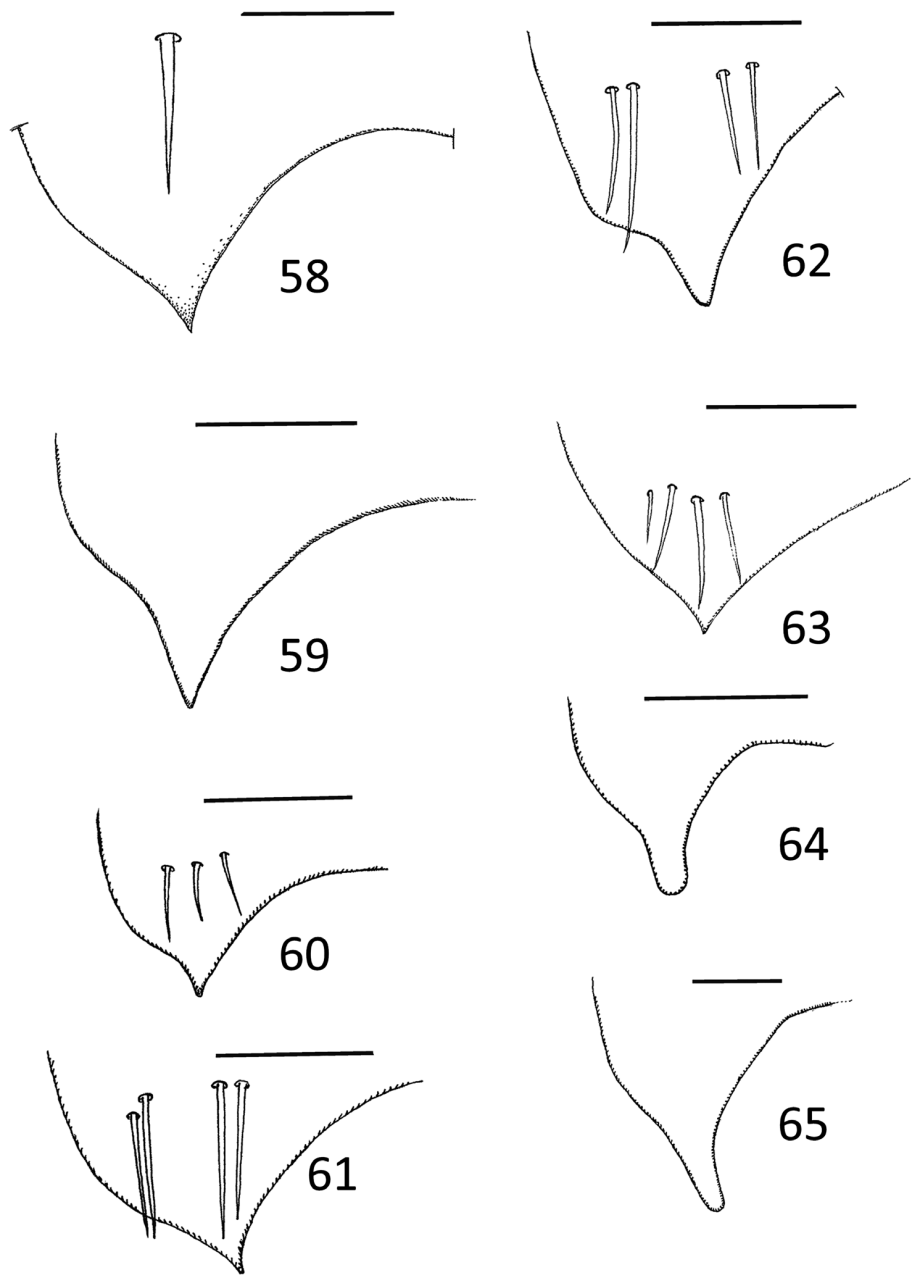
*Micronecta* spp., mediocaudal process of sternite VII, in ventral view: **58** *Micronecta decorata* Lundblad, 1933 **59** *Micronecta ludibunda* Breddin, 1905 **60** *Micronecta lumutensis* Chen, Nieser & Lansbury, 2008 **61** *Micronecta liewi* sp. n., paratype **62** *Micronecta lakimi* sp. n., paratype **63** *Micronecta skutalis* Nieser & Chen, 1999 **64** *Micronecta kymatista* Nieser & Chen, 1999 **65** *Micronecta quadristrigata* Breddin, 1905. Scale bars: 0.1 mm (**58–64**); 0.05 mm (**65**).

**Figures 66–73.**
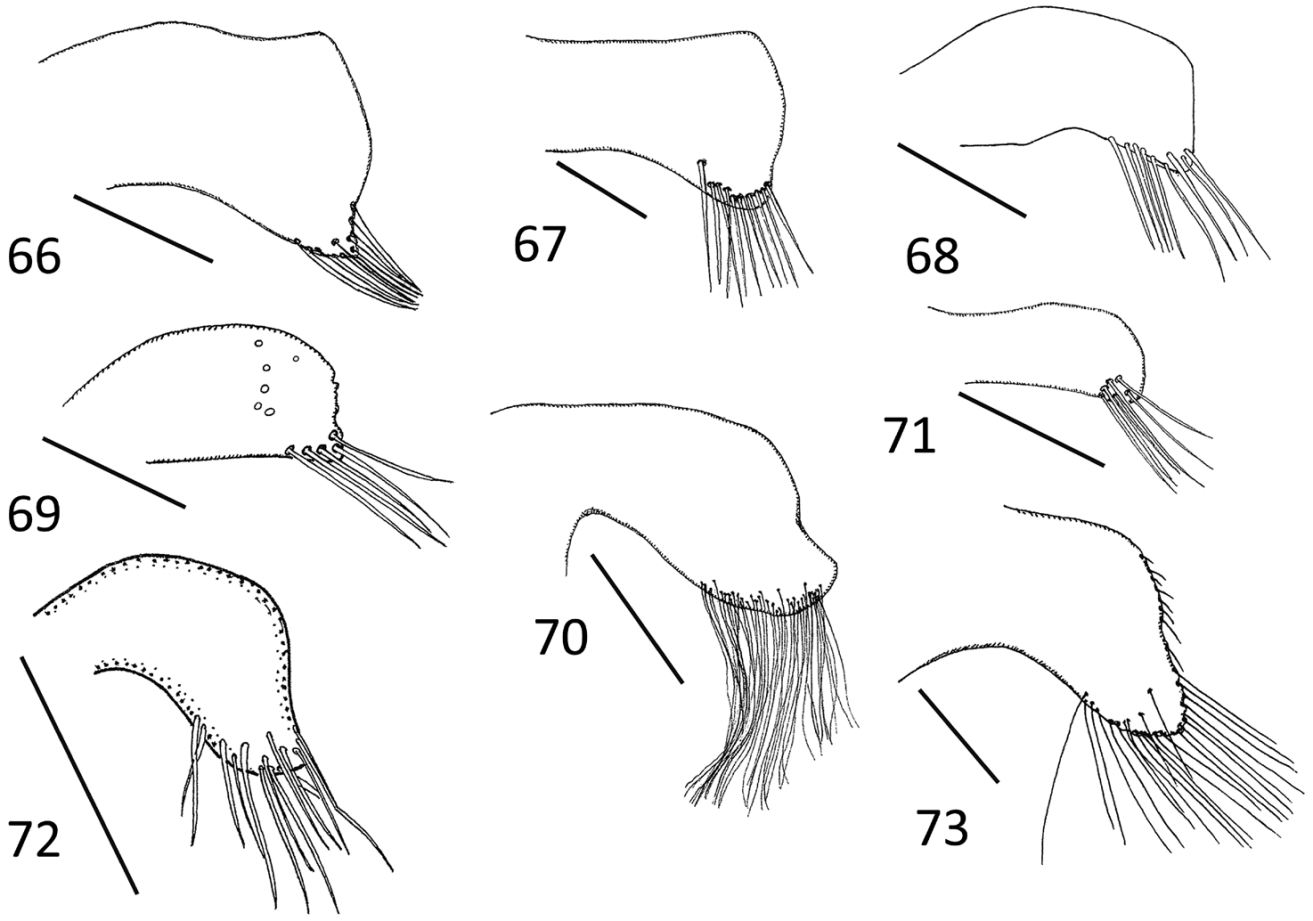
*Micronecta* spp., free lobe at right side of tergite VIII, in dorsal view: **66** *Micronecta decorata* Lundblad, 1933 **67** *Micronecta ludibunda* Breddin, 1905 **68** *Micronecta lumutensis* Chen, Nieser & Lansbury, 2008 **69** *Micronecta liewi* sp. n., paratype **70** *Micronecta lakimi* sp. n., paratype **71** *Micronecta skutalis* Nieser & Chen, 1999 **72** *Micronecta kymatista* Nieser & Chen, 1999 (scale 0.2 mm) **73** *Micronecta quadristrigata* Breddin, 1905. Scale bars: 0.01 mm (**66–71, 73**); 0.2 mm (**72**).

**Figures 74–89.**
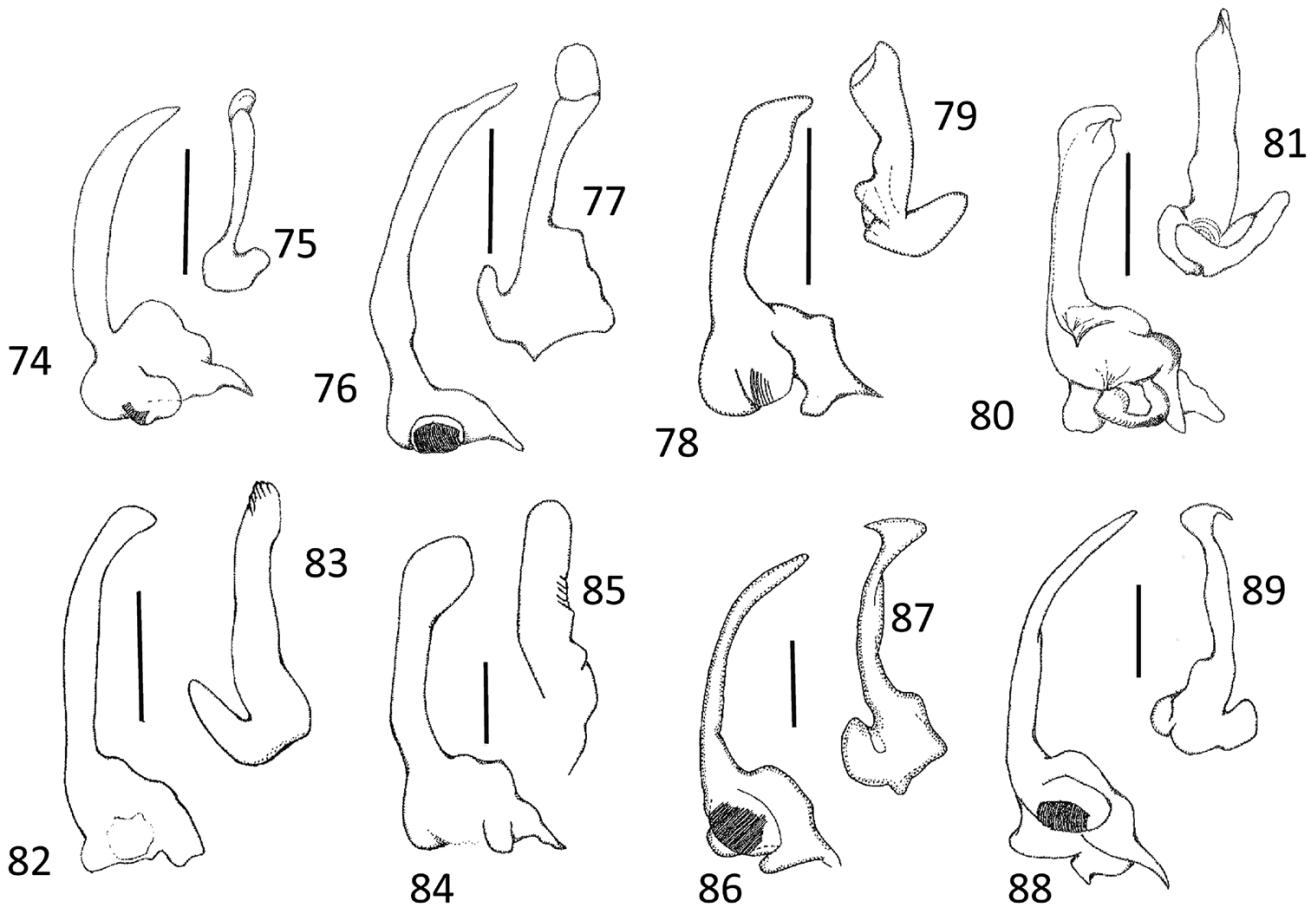
*Micronecta* spp., parameres: **74, 76, 78, 80, 82, 84, 86, 88:** right parameres in external view; **75, 77, 79, 81, 83, 85, 87, 89:** left parameres. **74–75** *Micronecta decorata* Lundblad, 1933 **76–77** *Micronecta ludibunda* Breddin, 1905 **78–79** *Micronecta lumutensis* Chen, Nieser & Lansbury, 2008 **80–81** *Micronecta lakimi* sp. n., paratype **82–83** *Micronecta liewi* sp. n., paratype **84–85** *Micronecta skutalis* Nieser & Chen, 1999 **86–87** *Micronecta kymatista* Nieser & Chen, 1999 **88–89** *Micronecta quadristrigata* Breddin, 1905. Scale bars: 0.1 mm.

*Female*. Fore femur with the same general arrangement of pegs and setae as in male. The seminal capsule of spermatheca mushroom-shaped (Fig. 91).

**Figures 90–97.**
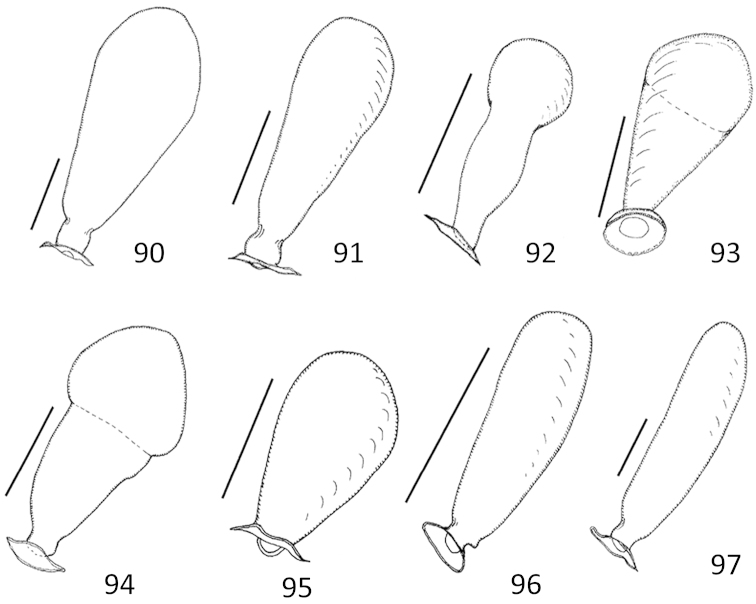
*Micronecta* spp., seminal capsule of spermatheca, in dorsal view: **90** *Micronecta decorata* Lundblad, 1933 **91** *Micronecta ludibunda* Breddin, 1905 **92** *Micronecta lumutensis* Chen, Nieser & Lansbury, 2008 **93** *Micronecta liewi* sp. n., paratype **94** *Micronecta lakimi* sp. n., paratype **95** *Micronecta skutalis* Nieser & Chen, 1999 **96** *Micronecta kymatista* Nieser & Chen, 1999 **97** *Micronecta quadristrigata* Breddin, 1905. Scale bars: 0.1 mm (**90–96)**; 0.05 mm (**97**).

#### Comparative notes.

Within Bornean *Micronecta*, this species is easily recognized in both sexes by its distinct linear pattern on the hemelytra (Figs 15, 22, 25).

#### Distribution.

This species with a wide distributional pattern, so far has been reported from: India and Sri Lanka (Hutchinson 1940, Wróblewski 1972), Thailand (Wróblewski 1968), Vietnam (Wróblewski 1967), West Malaysia (Leong 1966), Indonesia (Breddin 1905a, Lundblad 1933, Nieser and Chen 1999), New Guinea and Solomon Islands (Tinerella 2008), and introduced into Florida, U.S.A. (Polhemus and Golia 2006). Nieser and Chen (1999) mentioned one male from Borneo: Kalimantan Timur in NHMW.

### Subgenus Micronecta

Taxon classificationAnimaliaHemipteraMicronectidae

Kirkaldy, 1897

#### Type species.

*Notonecta minutissima* Linnaeus, 1758, by original designation.

#### Diagnosis.

Males with palar claw usually relatively large and apically dilated; sternite VIII with three to six (usually four) well-developed bristles; shaft of left paramere usually plate-like with subparallel margins.

### Micronecta (Micronecta) lumutensis

Taxon classificationAnimaliaHemipteraMicronectidae

Chen, Nieser & Lansbury, 2008

Figs 16 , 34 , 45 , 60 , 68 , 78 , 79 , 92

Micronecta lumutensis Chen, Nieser & Lansbury, 2008: 270–272 (original description).

#### Type material examined.

**INDONESIA: Kalimantan Timur:** Pasir, Gunung Lumut, 2 km E of Rantaulayong, 01°36.36'S, 115°58.38'E, 24.XI.2005, E. Gassó Miracle, EGM25, evergreen rainforest along river, at light, ML 19/21 hrs., 1 male holotype, 1 male and 2 female paratypes, all macropterous (RMNH).

#### Redescription.

Macropterous form. Generally a small (body length 1.5 mm) grayish *Micronecta*, with poorly contrasting markings.

*Dimensions*. Body length: male 1.48–1.52, female 1.45–1.50; width: male 0.69–0.72, female 0.52–0.54; diatone: male 0.49–0.51, female 0.52–0.54; width of pronotum: male 0.54–0.57, female 0.56–0.58; ocular index: male 1.77–1.78, female 1.59–1.60. Body length 2.1–2.5 times the maximal width. Pronotum slightly wider than head, synthlipsis wider than the posterior width of an eye (S/E 0.24/0.16).

*Colour*. Vertex sordid yellow, the frons yellowish with a brown spot, eyes grey, rostrum yellowish with dark brown transverse grooves. Pronotum yellowish brown, disk unmarked, posterior and lateral margins with a yellowish stripe. Hemelytra yellowish brown, apex of clavus darker brown, corium with three interrupted longitudinal brown stripes (Fig. 16), right membrane poorly delimited from the corium, with the same colour and texture as corium but without darker stripes, left membrane more distinctly separated from its corium, hyaline, and more membranous than the corium. Venter of abdomen and thorax grayish, legs yellowish.

*Pronotum* (Fig. 16) convex dorsally, about two and half times as wide as long (W/L male 0.56/2.1, female 0.57/0.23). Hemelytra smooth, beset with small spinules, notably on corium, arranged in longitudinal rows, and along the membranal suture. Spines laterally on abdominal segments: V with two short and one longer stout spines; VI with two or three short and one intermediate spine; VII with three or four short, one intermediate, and one or two long, stout spines; VIII with five short spines and two long hair-like bristles.

*Legs*. Length of leg segments: fore leg: male: femur 0.19, tibia 0.07, pala 0.11; female: femur 0.20, tibiotarsus 0.20; middle leg: male and female: femur 0.49, tibia 0.17, tarsus 0.24, claw 0.17; hind leg: male and female: femur 0.31, tibia 0.25, tarsus I 0.26, tarsus II 0.12, claw 0.07. Palmar bristles: about 15 in upper row, about 16 in lower row.

*Male*. Fore femur (Fig. 34) with a pair of pegs on proximal third, a subdistal peg dorsally, and one or two small pegs distally; pala with three long, dorsal hairs. Claw slender, clavate. Dorsum of abdomen: prestrigilar flap (Fig. 45) with a short, acute apex; strigil small, suboval, at a magnification of 400×, no separate teeth observable; free lobe of left part of tergite VIII (Fig. 68) more or less parallel-sided, softly curved, with a rounded apex and 9–10 apical bristles. Mediocaudal lobe of sternite VII (Fig. 60) short, acute, with three or four larger bristles. Left paramere (Fig. 79) apically slightly dilated, with an apical impression; right paramere in lateral view (Fig. 78) gradually widened toward apex, basal lobe with about eight stridulatory ridges.

*Female*. Fore femur with the same general arrangement of pegs and setae as in male. Seminal capsule of spermatheca mushroom-shaped (Fig. 92).

#### Comparative notes.

The small size, with a body length of about 1.5 mm, separates this species from other Bornean species except *Micronecta skutalis*. Males of *Micronecta lumutensis* and *Micronecta skutalis* can be separated by the characters of parameres as given in the key (Figs 78–79, 84–85). In addition, the seminal capsule of *Micronecta lumutensis* is mushroom-shaped (Fig. 92), whereas that of *Micronecta skutalis* is egg- or urn-shaped (Fig. 95). Females can be indentified only by their association with males.

#### Habitat.

The type specimens were collected at light in a mountainous area.

#### Distribution.

Indonesia: Kalimantan Timur (Chen et al. 2008).

### Micronecta (Micronecta) liewi sp. n.

Taxon classificationAnimaliaHemipteraMicronectidae

http://zoobank.org/49A37756-016D-4BF7-A0B3-FAE0BAF10C5F

Figs 17 , 26 , 35 , 36 , 46 , 53 , 61 , 69 , 82 , 83 , 93 , 98

#### Type material examined.

**Figure 98.**
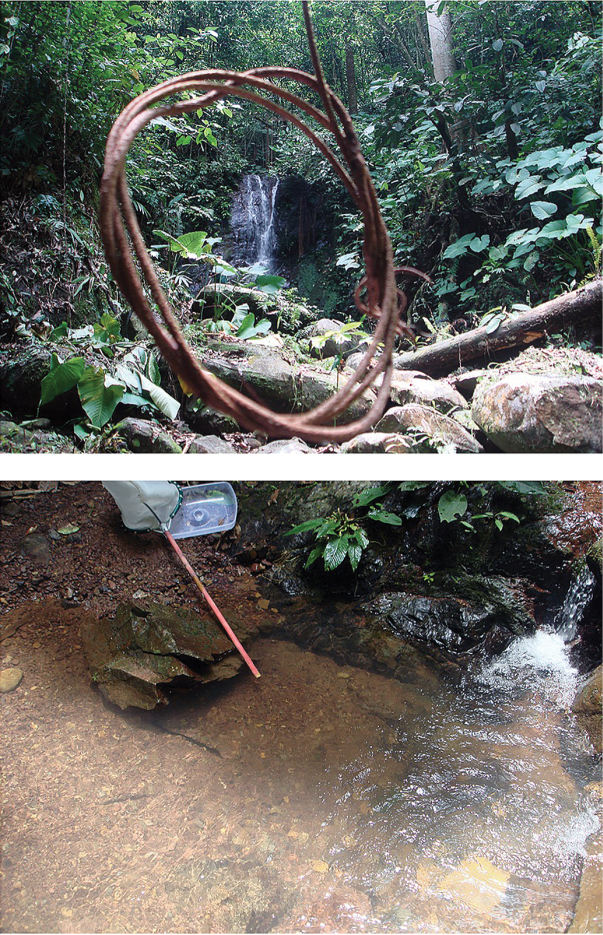
Waterfall in Sungai Kibabangan (above) at Substation Inobong, the Sabah Parks, Sabah Malaysia; downstream of the waterfall (below), where the specimens of *Micronecta liewi* sp. n. were collected.

Holotype: male (body length 1.72, in RMNH), MALAYSIA: Sabah, Crocker Range, Inobong Substation, Sungai Kibambangan (Fig. 98), downstream of waterfall, 05°51.28'N, 116°08.41'E, 433 m. a.s.l., 18.ix.2012, leg. P. Chen, N. Nieser & J. Lapidin, CN1277. Paratypes: same data as holotype, 12 males, 17 females. All macropterous (in RMNH, NCTN, NMPC, ZCSM).

#### Description.

Macropterous form (Fig. 17). Generally, a rather small (body length 1.7–1.8) yellowish to light brown species, with distinct brown markings.

*Dimensions*. Length: male 1.71–1.79, female 1.72–1.82; width: male 0.89–0.90, female 0.89–0.93; diatone: male 0.65–0.68, female 0.64–0.69; width of pronotum: male 0.71–0.71, female 0.70–0.75; ocular index: male 1.82–2.06, female 1.89–2.18. Body length twice maximal width (male 1.74/0.90, female 1.78/0.91).

*Colour*. Frons and vertex sordid yellow, eyes dark castaneous. Pronotum and hemelytra sordid yellow to light brown, the hemelytra with a broad transverse medium to dark brown band at middle (Fig. 26), left membrane medium to dark brown. Disk of pronotum unmarked. Venter and thorax sordid yellow, laterally infuscate, abdomen grayish brown, medially variably lighter. Legs pale yellow.

*Head* slightly narrower than pronotum, synthlipsis 1.7–1.8 times as wide as the posterior margin of an eye.

*Pronotum* well developed, dorsally convex with lateral margins distinctly straight, and more or less truncate (Fig. 17), slightly over 2.5 times as wide as long (W/L male 0.71/0.26, female 0.72/0.28). Hemelytra (Fig. 26) smooth, beset with extremely small unobtrusive spinules. Spines laterally on abdominal segments: V with one short and one long spine, VI with two short, and two long spines; VII with three or four short and one long spine; VIII with four or five medium long spines and two long hair-like bristles.

*Legs*. Length of leg segments: fore leg: male: femur 0.24, tibia 0.11, pala 0.12; female: femur 0.23, tibiotarsus 0.22; middle leg: male: femur 0.54, tibia 0.17, tarsus 0.25, claw 0.14; female: femur 0.56, tibia 0.19, tarsus 0.23, claw 0.15; hind leg: male: femur 0.39, tibia 0.29, tarsus I 0.32, tarsus II 0.13, claw 0.08; female: femur 0.42, tibia 0.33, tarsus I 0.32, tarsus II 0.13, claw 0.08. Palmar bristles: about 13 in lower row and ca. 11 in upper row.

*Male*. Fore femur (Fig. 35) with a pair of pegs in proximal third, one peg dorsally at distal third and two pegs dorsodistally; tibia without dorsoapical peg; pala with three comparatively short dorsal hairs, 10–12 short bristles in upper row, distal bristle of upper row much stouter and longer than other upper bristles, and 14 to 16 longer bristles in lower row. Claw simple, elongate (Fig. 36). Dorsum of abdomen: prestrigilar lobe (Fig. 46) with a pointed apex, strigil small and narrow (Fig. 53), comb with about 75 teeth, free lobe of left part of tergite VIII (Fig. 69) caudally truncate. Left paramere (Fig. 83) with a wide shaft, apex with short longitudinal grooves; right paramere (Fig. 82) with a medium-sized shaft and a slightly expanded apex, basal lobe strongly developed, stridulatory ridges not observed. Mediocaudal lobe of sternite VII (Fig. 61) with four bristles.

*Female*. Fore femur with the same general arrangement of pegs and setae as in male. Seminal capsule of spermatheca urn-shaped (Fig. 93).

#### Comparative notes.

The hemelytral pattern is diagnostic among the Melanesian *Micronecta* fauna. *Micronecta liewi* is similar to *Micronecta melanopardala melanopardala* Nieser & Chen, 2003 described from the Philippines by having a similar transverse band midway along the hemelytra, but it differs from *Micronecta melanopardala melanopardala* by lacking a dark patch on the clavi as in *Micronecta melanopardala*. In general, *Micronecta liewi* has more distinct dark markings than in *Micronecta melanopardala adiaphana* Nieser & Chen, 2003. Furthermore, in both subspecies of *Micronecta melanopardala*, the shafts of the right parameres are more slender than *Micronecta liewi*, and the apex of the right paramere of *Micronecta melanopardala* is not expanded.

The strongly developed distal bristle of the upper row on the male pala (Figs 35, 36) gives impression of an additional claw as in the subgenus *Unguinecta* Nieser, Chen & Yang, 2005 from southern continental Asia. However, the four bristles on mediocaudal lobe of sternite VII of the male, the shape of the parameres, and the shape of the free lobe on the left part of tergite VIII of the male will all allow placement in the subgenus *Micronecta*.

#### Etymology.

This species is named in honor of Dr. Thor Seng Liew (NBC Naturalis and Sabah University, Malaysia), for his outstanding contributions to the study of the biodiversity of Sabah, and his invaluable help with our work on water bugs in Borneo.

#### Habitat.

The type series was collected in a small, virtually stagnant bay on the downstream side of Kibambangan waterfall (Fig. 98).

#### Distribution.

Malaysia: Sabah (this paper).

### Micronecta (Micronecta) lakimi sp. n.

Taxon classificationAnimaliaHemipteraMicronectidae

http://zoobank.org/F8B54026-F285-47D7-954F-8D522FDA38EB

Figs 18 , 27 , 37 , 38 , 47 , 54 , 62 , 70 , 80 , 81 , 94 , 99

#### Type material examined.

**Figure 99.**
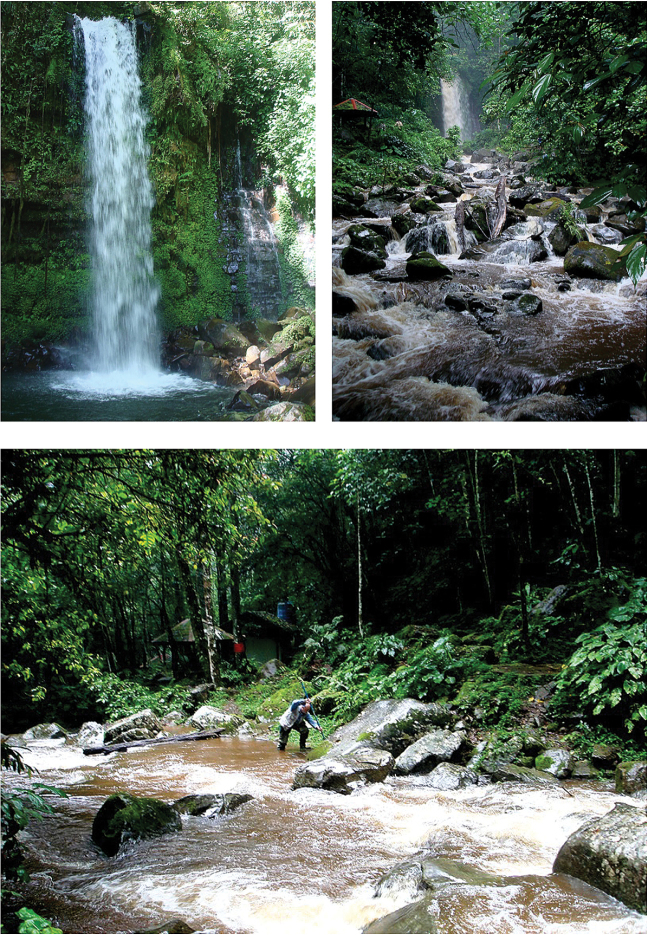
Waterfall Mahua (above) in Mahua Sub-station, The Sabah Parks, Sabah, Malaysia; downstream of the waterfall (below), one of the sites where *Micronecta lakimi* was collected.

Holotype: male (body length 1.00 mm, in RMNH), MALAYSIA: Sabah, Kota Belud Dist., Crocker Range, Mahua Substation, Mahua waterfall (fig. 99), 05°47.59'N, 116°24.08'E, 1215 m. a.s.l., 21.IX. 2012, leg. P. Chen, N. Nieser & J. Lapidin, CN1281. Paratypes: same data as holotype, 7 males, 25 females; MALAYSIA: Sabah, Kota Belud Dist., Crocker Range Park, Sungai Mahua near entrance of Mahua Substation, 05°47.53'N, 116°24.19'E, 1053 m. a.s.l., 22.ix.2012, leg. P. Chen, N. Nieser & J. Lapidin, CN1283, 10 males, 3 females. (Paratypes in RMNH, NCTN, NMPC, ZCSM).

#### Description.

Macropterous form (Fig. 18). Generally a medium-sized (body length 2.1–2.2), rather dark grayish-brown species, without obvious markings.

*Dimensions*. Length: male 2.07–2.22, female 2.11–2.13; width: male 0.92–1.00 female 1.01–1.04; diatone: male 0.74–0.76, female 0.75–0.77; width of pronotum: male 0.83–0.88, female 0.84–0.88; ocular index: male 1.57–1.89, female 1.76–2.05. Body length slightly over twice maximal width (male 2.16/0.97, female 2.12/1.02). Head in dorsal view short, its median length less than half the median length of pronotum (male 0.14/0.33, female 0.15/0.36). Head narrower than pronotum, synthlipsis 1.5–1.7 times as wide as the posterior margin of an eye (male 0.35/0.23, female 0.37/0.22).

*Colour*. Vertex yellowish, with a small dark brown point at middle of posterior margin (raised for air intake), eyes grayish. Pronotum unicolorous, medium-brown except for a narrow yellow transverse band along posterior margin. Scutellum reddish brown. Hemelytra medium brown, clavus with a reddish stripe along the scutellar margin, pruinose area at base of embolar groove black, apical third of corium light brown, laterally with a reddish tinge. Frons medium brown, rostrum with a dark median gray marking. Thoracic and abdominal venter dull dark grayish to blackish. Legs pale yellow, anterior femur with a brownish stripe and intermediate tarsus I with a small black spot distally.

*Pronotum* well developed, dorsally convex with lateral margins distinctly truncate (Fig. 18), about 2.5 times as wide as long (W/L male 0.85/0.34, female 0.87/0.36). Hemelytra (Fig. 27) smooth, beset with small, distinct spinules, most notably on corium. Spines laterally on abdominal segments: V with three short and one long spine; VI with two short and two long spines; VII with two short and two long spines; VIII with four or five short spines and two long hair–like bristles.

*Legs*. Length of leg segments: fore leg: male: femur 0.27, tibia 0.13, pala 0.14; female: femur 0.26, tibiotarsus 0.26; middle leg: male: femur 0.66, tibia 0.20, tarsus 0.37, claw 0.21; female: femur 0.65, tibia 0.23, tarsus 0.36, claw 0.21; hind leg: male: femur 0.49, tibia 0.35, tarsus I 0.38, tarsus II 0.16, claw 0.10; female: femur 0.46, tibia 0.38, tarsus I 0.38, tarsus II 0.16, claw 0.10. Palm of pala with about 14 bristles in upper row and about 17 in lower row.

*Male*. Fore femur (Fig. 37) with a pair of pegs on proximal third and one peg dorsodistally; tibia without dorsoapical peg; pala with three comparatively short dorsal hairs. Claw simple, dilated distally (Fig. 38). Dorsum of abdomen: prestrigilar flap (Fig. 47) with a elongate, weakly acute apex; strigil comparatively large, comb (Fig. 54) narrow, with about 75 teeth; free lobe of left part of tergite VIII (Fig. 70) with a somewhat sinuate apex with about 30 bristles. Left paramere (Fig. 81) with a wide, roughly parallel-sided shaft, apex abruptly narrowed; right paramere (Fig. 80) with a medium-sized shaft and an expanded apex with a short finger–like projection; basal lobe well developed, with 25 stridulatory ridges. Mediocaudal lobe of sternite VII (Fig. 62) with four bristles.

*Female*. General arrangement of bristles on fore femur is the same as in male. The seminal capsule of spermatheca mushroom–shaped (Fig. 94).

#### Comparative notes.

The right paramere is apically somewhat similar to that of *Micronecta ornitheia* Nieser et al., 2005 from Yunnan, China. However, the shaft of the right paramere of *Micronecta orniteia* is narrower, the left paramere is apically truncate; and it is a smaller species; body length of *Micronecta orniteia* is 1.7–1.9, body length of *Micronecta lakimi* is 2.1–2.2.

#### Etymology.

The species is named after Dr. Maklarin Lakim for his great service organizing the joint expedition to Sabah Parks in 2012, and his various activities in support of biodiversity exploration in Sabah Parks.

#### Habitat.

The type series was collected downstream of Mahua waterfall, at the edge of the stream with a slow current (Fig. 99).

#### Distribution.

Malaysia: Sabah (this paper).

### Micronecta (Micronecta) skutalis

Taxon classificationAnimaliaHemipteraMicronectidae

Nieser & Chen, 1999

Figs 19 , 28 , 39 , 40 , 48 , 55 , 63 , 71 , 84 , 85 , 95

Micronecta skutalis Nieser & Chen, 1999: 86–87 (original description).

#### Type material examined.

Holotype macropterous male (RMNH), MALAYSIA: Sabah, 60 km W of Lahad Datu, Danum Valley Field Centre at junction of Sungai Segama and Sungai Palum Tambun, bridge of Segama, 4°58'N, 117°48'E, 750m a.s.l., edge of untouched lowland rainforest, 14 march 1987, at light, 18.20–22.30h leg. Van Tol & Huisman. Paratypes, same data as holotype 12male 11 females (RMNH).

#### Additional material examined.

**MALAYSIA: Borneo: Sabah:** 60 km West of Lahad Datu: Danum Valley Field Centre, at junction of Sungai Segama and Sungai Palum Tambun, 4°58'N, 117°48'E, 150 m a.s.l., 14.iii.1987, 18.20–22.30 hr., edge of untouched evergreen lowland forest, leg. J. van Tol & Huisman, 5 males, 14 females. (RMNH, 2 males, 2 females NCTN); 75 km West of Lahad Datu, confl. S. Sabran, S. Danum, S/N, 4°57'N, 117°41'E, 200 m, 23.x.1987, leg. J. Huisman & R. de Jong, 1 male, 2 females; 10 km SE of Ranau, Kg. Nalapak, Sungai Kananapun, 5°58'N, 116°47'E, 7.ii.1987, leg. J. Huisman, 2 females (RMNH). All macropterous, collected at light.

#### Redescription

(based on dry specimens mounted on carton). Macropterous form (Fig. 19). A small (length 1.5–1.7 mm), light to medium-brown species; hemelytra smooth, with a variable number of small pegs scattered over their surface.

*Dimensions*. Length, male 1.52–1.57, female 1.53–1.70; width, male 0.63–0.70, female 0.62–0.70; diatone, male 0.53–0.56, female 0.51–0.55; width of pronotum, male 0.56–0.61, female 0.57–0.59; ocular index, male 1.44–1.61, female 1.57–1.86. Body length 2.3–2.6 the maximal width. Pronotum slightly wider than head, synthlipsis wider than the posterior width of an eye (S/E 0.23/0.17).

*Colour*. Frons and vertex sordid yellow, eyes grayish. Pronotum and hemelytra sordid yellow to light brown; hemelytra with an often indistinct, transverse medium to dark brown band at middle (Fig. 28), left membrane medium to dark brown. Venter: thorax sordid yellow, laterally infuscate; abdomen grayish brown, medially variably lighter. Legs pale yellow.

*Pronotum* dorsally convex, 2–2.5 times as wide as long (W/L 2.1–2.7, Fig. 19). Hemelytra beset with spinules arranged in longitudinal rows and along membranal suture. Spines laterally on abdominal segments: V with two short and one long stout spine; VI with two short and one or two longer spines; VII with two short and two long stout spines; VIII with five short spines and two long hair-like bristles.

*Legs*. Length of leg segments: fore leg: male: femur 0.31, tibia 0.15, pala 0.21; female: femur 0.32, tibiotarsus 0.32; middle leg: male: femur 0.53, tibia 0.19, tarsus 0.26, claw 0.19; female: femur 0.50, tibia 0.18, tarsus 0.26, claw 0.15; hind leg: male: femur 0.32, tibia 0.28, tarsus I 0.25, tarsus II 0.12, claw 0.09; female: femur 0.33, tibia 0.29, tarsus I 0.27, tarsus II 0.12, claw 0.09. Palmar bristles 15–19 in upper row, about 14–17 in lower row.

*Male*. Fore femur (Fig. 39) with a pair of pegs on proximal third, and two or three small pegs distally; tibia with two to three small spines near dorsal margin; pala (Fig. 40) with three long dorsal hairs. Claw simple, clavate. Dorsum of abdomen: prestrigilar flap (Fig. 48) with a short apex; strigil (Fig. 55) small, sub-oval, one comb with about 60 teeth; free lobe of left part of tergite VIII (Fig. 71) more or less parallel-sided, softly curved, with a rounded apex and about10 apical bristles. Mediocaudal lobe of sternite VII (Fig. 63) short, acute, with four larger bristles. Left paramere (Fig. 85) parallel-sided, apically rounded, with an indentation at the base of the shaft; right paramere in lateral view (Fig. 84) apically dilated, basal lobe with about eight stridulatory ridges.

*Female*. Fore femur with the same general arrangement of pegs and setae as in male. Seminal capsule of spermatheca ovate (Fig. 95).

#### Comparative notes.

Its small size separates this species from other Bornean species of *Micronecta*, except for *Micronecta lumutensis* (see that species).

#### Habitat.

The specimens all have been collected at light near a stream.

#### Distribution.

Malaysia: Sabah (Nieser and Chen 1999).

### Subgenus Sigmonecta

Taxon classificationAnimaliaHemipteraMicronectidae

Wróblewski, 1962

#### Type species.

*Micronecta quadristrigata* Breddin, 1905, by monotypy.

#### Diagnosis.

Medium-sized to larger *Micronecta*, body length 2.2–3.2 mm. Males with process of abdominal sternite VII elongate, tongue-like, with a rounded tip (Figs 64, 65), and without larger bristles; strigil present; free lobe of tergite VIII sigmoid (Fig. 72); and left paramere with a sickle-shaped apex (Figs 87, 89).

#### Remarks.

Wróblewski (1962: 176) erected *Sigmonecta* as a new subgenus for *Micronecta quadristrigata* Breddin, 1905, without describing the subgenus. His comments were as follows: “I have already stressed in an earlier paper (Wróblewski 1960a) the isolated systematic position of *Micronecta quadristrigata* Bred. Now I propose to place it in a separate, so far monotypic subgenus Sigmonecta subg. n., named so on account of the sigmoid outline of the eighth abdominal tergite in the males.”

### Micronecta (Sigmonecta) kymatista

Taxon classificationAnimaliaHemipteraMicronectidae

Nieser & Chen, 1999

Figs 8 , 20 , 41 , 49 , 56 , 64 , 72 , 86 , 87 , 96

Micronecta kymatista Nieser & Chen, 1999: 82–83 (original description).

#### Type material examined.

Holotype macropterous male (RMNH), **INDONESIA: Sulawesi Utara**, Dumoga Bone N.P. Malibagu Road 10 km H, ca. 250m a.s.l., 2 sept.1985, secondary growth, at light, leg. J. Huijbregts, HH437. Paratypes, same data as holotype, 14 males, 16 females (RMNH).

#### Additional Material examined.

**INDONESIA: Sulawesi Utara:** Dumoga Bone N.P., Malibagu Road, 10 km N, ca. 250 m a.s.l., 2.ix.1985, second growth, at light, leg. J. Huijbregts, 1 female. **Sulawesi Tenggara:** Wawonggole, Sungai Anggoro, 20.ii.1989, sluggish stream in open woodland, leg. N. Nieser, N8801, 1 female; **Sulawesi Tenggara:** Desa Kagunyala, pond overgrown by *Azolla* and *Lemna*, 21.ii.1989, leg. N. Nieser, N8906, 1 male; **Sulawesi Tenggara:** Pulau Buton, mangrove swamp along road Bau-bau to Lawele, 9.iii.1989, leg. N. Nieser, 2 males (all macropterous paratypes, in NCTN).

#### Redescription.

Macropterous form. Generally a quite large (body length 2.8–3.1), light to medium-brown species; corium with four longitudinal, brownish stripes, very often interrupted.

*Dimensions*. Length: male 2.8–2.9, female 2.9–3.1; width: male 1.25–1.32, female 1.28–1.39; diatone: male 1.01–1.03, female 1.04–1.11; width of pronotum: male 0.98–1.01, female 1.02–1.08; ocular index: male 1.25–1.32, female 1.17–1.30. Body length 2.15 times maximal width (male 2.46/1.10, female 2.79/1.22). Head slightly wider than pronotum (male 1.02/1.00, female 1.08/1.05), synthlipsis 1.2 times as wide as the posterior margin of an eye.

*Colour*. Frons and vertex sordid yellow, eyes grayish. Pronotum light to medium brown, disk unmarked, posterior margin with a distinct yellowish stripe. Hemelytra sordid yellow to light brown, clavus with a darker medium-brown stripe along the suture between clavus and corium suture, corium typically with four fragmented longitudinal medium-brown stripes (Fig. 20), embolium yellowish with three or four indistinct brownish spots; right membrane poorly delimited from the corium, with the same colour and texture but without darker stripes; left membrane more distinctly separated from corium, hyaline to somewhat smoky and more membranous than the corium. Venter, abdomen, thorax, and legs pale yellow.

*Pronotum* well developed, dorsally convex with lateral margins straight or more or less truncate (Fig. 20), about three times as wide as long (W/L male 1.00/0.34, female 1.05/0.36). Hemelytra smooth, beset with numerous small but distinct spinules. Spines laterally on abdominal segments: V with two short and one longer stout spine; VI with two or three short and one long spine; VII with two or three short and one long stout spine; VIII with five or six short and one longer, stout spine and two long hair-like bristles.

*Legs*. Length of leg segments: fore leg: male: femur 0.26, tibia 0.14, pala 0.14; female: femur 0.26, tibiotarsus 0.26; middle leg: male: femur 0.70, tibia 0.23, tarsus 0.30, claw 0.25; female: femur 0.76, tibia 0.23, tarsus 0.33, claw 0.26; hind leg: male: femur 0.46, tibia 0.36, tarsus I 0.40, tarsus II 0.13, claw 0.08; female: femur 0.48, tibia 0.37, tarsus I 0.44, tarsus II 0.16, claw 0.08. Palmar bristles: 15 in upper and lower row.

*Male*. Fore femur with a pair of pegs on proximal third, and a pair of small pegs distally; tibia with a dorsoapical peg. Pala (Fig. 41) with three long dorsal hairs, the apical bristles in lower row distinctly thicker than the bristles of lower row. Claw broadly clavate, gradually dilated from base to apex, without ventral notch. Dorsum of abdomen: prestrigilar lobe (Fig. 49) difficult to observe, strigil (Fig. 56) with one, relatively broad comb with about 50 elongate teeth. Median lobe of sternite VII (Fig. 64) apically narrow with a rounded apex, without obvious longer bristles. Free lobe of left part of tergite VIII (Fig. 72) sigmoid with about 12 apical bristles. Medial margin of right lobe of tergite VIII with 28–35 bristles caudally, placed in a double to triple row on caudal half (Fig. 8). Left paramere (Fig. 87) with a comparatively narrow shaft and a sickle-shaped apex; right paramere, in lateral view (Fig. 86), with an evenly curved, more or less parallel-sided, apically tapering shaft, basal lobe with about 40 stridulatory ridges on the pars stridens.

*Female*. Fore leg with the same general arrangement of pegs and setae as in male. Seminal capsule of spermatheca elongate-clavate (Fig. 96).

#### Comparative notes.

This species is similar to *Micronecta quadristrigata*, which is smaller on average and has fewer bristles on the caudal half of inner margin of right part of tergite VIII in males (see key and Figs 8–9).

#### Habitat.

This species has been found in ponds and sluggish streams mostly in less disturbed areas.

#### Distribution.

Indonesia: Sulawesi and Borneo (Kalimantan Timur) (Nieser and Chen 1999).

### Micronecta (Sigmonecta) quadristrigata

Taxon classificationAnimaliaHemipteraMicronectidae

Breddin, 1905 new record for Borneo

Figs 5 , 9 , 21 , 23 , 29 , 42 , 50 , 57 , 65 , 73 , 88 , 89 , 97

Micronecta quadristrigata Breddin, 1905a: 57 (original description).
Micronecta quadristrigata : Breddin 1905b: 156–157 (extensive description).
Micronecta quadristrigata : Lundblad 1933: 87–191 (redescription).
Micronecta quadristrigata : Wróblewski 1960: 301–304 (additional distributional and morphological notes).
Micronecta (Sigmonecta) quadristrigata : Wróblewski 1962: 176 (introducing subgenus).
Micronecta quadristrigata : Wróblewski 1968: 776 (checklist).
Micronecta quadristrigata : Wróblewski 1972: 29–133 (redefinition of species).
Micronecta (Sigmonecta) quadristrigata : Jansson 1995: 34 (catalogue).
Micronecta quadristrigata Cassis & Goss, 1995: 69 (distribution in Australia)
Micronecta quadristrigata : Nieser and Chen 1999: 80 [recorded from Indonesia (Sulawesi) and Philippines (Mindanao)].
Micronecta quadristrigata : Chen et al. 2005: 420 (checklist).
Micronecta quadristrigata : Tinerella 2008: 39–145 [distribution in New Guinea Island, record from Indonesia (Moluccas)].
Micronecta (Sigmonecta) quadristrigata : Linnavuori et al 2011: 77–178 (record from United Arab Emirates).
Micronecta quadristrigata : Tinerella 2013: 102 (redescription, additional records in Australia).
Micronecta (Sigmonecta) quadristrigata For a discussion on the status of *Micronecta minthe* Distant, 1911, which is considered by some authors as a subspecies or synonym of *Micronecta quadristrigata*, see Jansson (1995) and Wróblewski (1972a).

#### Type material examined.

Syntype, INDONESIA: “Kotype; Djokjokarta (= Yogyakarta), **Java**, K. Kraepelin; leg. 18.III.1904, ded. 8.VI.1904; Breddin determ.; Lundblad revid. 1934”, 1f (ZMUH); syntype, INDONESIA: “Kotype; Buitenzorg (= Bogor)”, 1m 1f (ZMUH).

#### Additional material examined.

MALAYSIA: Sabah: Kota Belud Dist., Mt. Kinabalu, pond at Kampong Kiau, 06°01.48'N, 116°29.14'E, 1003 m a.s.l., 15.ix.2012, leg. P. Chen, N. Nieser & J. Lapidin, CN1273, 9 males, 15 females; Sabah, Kota Belud Dist., Mt. Kinabalu, Kota Belud, Head Quarter of Kinabalu Park, tributary of Sungai Kadamaian, 06°02.09'N, 116°29.39'E, 1410 m. a.s.l., 16.ix.2012, leg. P. Chen, N. Nieser & J. Lapidin, CN1275, 2 males; all macropterous (NCTN).

#### Redescription.

Macropterous form. Generally a medium-sized to quite large (body length reported 2.2–3.2, most specimens 2.5–3.0), yellowish to light-brown species, with four variable, indistinct, longitudinal, brown stripes on corium.

*Dimensions*. Length: male 2.2–2.9, female 2.5–3.2; width: male 1.07–1.15, female 1.12–1.37; diatone: male 0.83–1.12, female 0.87–1.18; width of pronotum: male 0.82–1.11, female 0.86–1.17; ocular index: male 1.20–1.55, female 1.17–2.16. Body length two and a quarter times maximal width (male 2.46/1.10, female 2.79/1.22). Head slightly wider than pronotum (male 0.89/0.88, female 0.99/0.98), synthlipsis 1.4–1.5 times as wide as the posterior margin of an eye.

*Colour*. Frons and vertex sordid yellow, eyes grayish. Pronotum light brown, virtually unmarked in most specimens, in some specimens, with two indistinct, usually interrupted transverse stripes, posterior margin with a poorly defined yellowish stripe. Hemelytra sordid yellow to light brown, clavus with a darker medium-brown stripe along the claval suture, and a smaller medium-brown streak near the inner angle; corium typically with four interrupted, longitudinal, medium- brown stripes (Figs 21, 23, 29), embolium with four black spots; right membrane poorly delimited from the corium, with the same colour and texture but without darker stripes; left membrane more distinctly separated from corium, hyaline and more membranous than the corium. Venter, thorax, and legs pale yellow, abdomen yellowish to light brown.

*Pronotum* well developed, dorsally convex with lateral margins straight or more or less truncate (Fig. 21), slightly over 2.5 times as wide as long (W/L male 0.88/0.34, female 0.98/0.37). Hemelytra smooth, beset with numerous small but distinct spinules. Spines laterally on abdominal segments: V with three short and one longer stout spine; VI with two short and two long spines; VII four short and one long stout spine; VIII with six short to longer, stout spines and one long hair-like bristle.

*Leg*. Length of leg segments: fore leg: male: femur 0.38, tibia 0.16, pala 0.16; female: femur 0.38, tibiotarsus 0.36; middle leg: male: femur 0.89, tibia 0.26, tarsus 0.38, claw 0.34, female; femur 0.98, tibia 0.28, tarsus 0.41, claw 0.3; hind leg: male: femur 0.58, tibia 0.42, tarsus I 0.42, tarsus II 0.19, claw 0.10; female: femur 0.62, tibia 0.46, tarsus I 0.46, tarsus II 0.21, claw 0.12. Palmar bristles: 14 to 15 in upper row, 11 to 12 in lower row.

*Male*. Fore femur (Fig. 42) with a pair of pegs in proximal third, two (in some specimens only one) small pegs about midway dorsally and a small peg dorsodistally; tibia with a larger peg subventrally on apical third and two small dorsoapical pegs; pala with four long dorsal hairs, distal bristle of lower row much stouter and longer than other lower bristles. Claw plump, clavate. Dorsum of abdomen: prestrigilar lobe with a short, broadly rounded apex, strigil (Fig. 57) sub-oval, comb with about 25 long teeth, free lobe of left part of tergite VIII (Fig. 73) sigmoid-shaped. Left paramere (Fig. 89) with a wide shaft, apex sickle-shaped; right paramere in lateral view (Fig. 88) with an evenly curved shaft, basal lobe strongly developed with about 50 stridulatory ridges; in dorsolateral view, the shaft is somewhat sinuous. Mediocaudal lobe of sternite VII (Fig. 65), long, with apical part elongate and obtusely rounded apically, without larger bristles.

*Female*. Fore femur with the same general arrangement of pegs and setae as in male. The seminal capsule of spermatheca elongate-clavate (Fig. 97).

#### Notes.

*Micronecta quadristrigata* might have an even broader range of size variation. Wróblewski (1960a) reported that females had a length up to 3.3 mm from Hong Kong, and Leong (1966) measured females with a length up to 3.4 mm from the Malay Peninsula. However, we have never seen specimens with a length over 3.1 mm.

#### Comparative notes.

See discussion under *Micronecta kymatista*.

#### Habitat.

Various stagnant and slowly flowing waters, especially in agricultural fields, including rice fields.

#### Distribution.

Widely spread through South and Southeast Asia to Hong Kong and Taiwan, and through Indonesia to the Philippines, New Guinea, and N. Australia; United Arab Emirates (Linnavuori et al 2011), Iran (Wróblewski 1960), India (Hutchinson 1940), Sri Lanka (Wróblewski 1964), Thailand (Wróblewski 1972), Vietnam (Wróblewski 1962), southern China, including Taiwan (Wróblewski 1968, 1972), West Malaysia (Leong 1966), Indonesia (Breddin 1905a, Lundblad 1933, Nieser and Chen 1999, Tinerella 2013), Philippines (Polhemus and Reisen 1976, Nieser and Chen 1999), New Guinea (Tinerella 2008), and Australia (Cassis and Gross 1995, Tinerella 2013).

## Discussion of faunistic components in Borneo

All Bornean species of Micronectidae belong to the dominant genus *Micronecta* Kirkaldy, 1897, which contains about 130 described species. The present knowledge of the Bornean fauna (and the Malesian fauna as a whole) of Micronectidae is still insufficient to discuss its proper biogeographical affinities. Judging from the literature, lowland species, such as *Micronecta decorata*, *Micronecta ludibunda*, *Micronecta quadristrigata*, tend to be more widespread than species from hilly areas, such as *Micronecta lakimi*, *Micronecta liewi*, and *Micronecta lumutensis*. This conclusion might be partly artificial because most taxa of Micronectidae are collected at light. They easily escape from the casual collector in the field due to their small size. The shallow stagnant waters in lowland ponds and marginal bays of streams are less stable than the stagnant waters in hills or mountains, such as a pond at the foot of a waterfall. Moreover, lowland species were found several times in very high densities, whereas species from hilly areas were always found in moderate to low densities. We hypothesize that lowland species more often colonize new habitats and therefore fly more often.

Of the eight species of *Micronecta* known from Borneo, three are so far endemic to the island: *Micronecta lakimi*, *Micronecta liewi*, and *Micronecta lumutensis*. Their localities are all in mountainous areas. It is unclear which species are closely related to *Micronecta lakimi* and *Micronecta liewi*. But *Micronecta lumutensis* apparently is closely related to *Micronecta skutalis*, which was described from Sabah and also has been found on Palawan in the Philippines (Nieser and Chen 2003). Another species related to *Micronecta lumutensis* and *Micronecta skutalis* is *Micronecta abra* Nieser & Chen, 2003 described from Palawan. These three species apparently constitute a species-group by having small body size, and each of them has limited distributional range around Borneo.

*Micronecta quadristrigata* is a widespread species. In the west, it reaches the United Arab Emirates and southern Iran (Linnavuori et al. 2011, Wróblewski 1960). The area around the Gulf of Persia and the Gulf of Oman is considered to belong to the Palaearctic Region (Aukema and Rieger 1995), but for water bugs it has a strong Oriental element as well as some species of African origin (Linnavuori et al. 2011). Besides the water bugs, the water beetles also show Oriental elements in Arabian Peninsula. Hájek and Wewalka (2009) stated: “Our study further reiterates that the Arabian Peninsula is a typical transition area between the neighboring major zoogeographical regions”. We agree with their observations based on the recent studies by different authors of insect fauna in the Arabian Peninsula, which has emphasized an interesting point from a zoogeographical point of view.

Eastward, *Micronecta quadristrigata* reaches New Guinea and northern Australia (Tinerella 2013), indicating that this species probably originated in the Oriental origin. The open and shallow man-made waters, such as rice fields, provide conditions that have allowed micronectid to spread westward and eastward.

*Micronecta kymatista* is closely related to *Micronecta quadristrigata*, but according to the locality information from Sulawesi and Borneo, it seems to prefer habitats somewhat less influenced by human activities. This might also explain the wide distribution of the other two lowland species *Micronecta decorata* and *Micronecta ludibunda*. It is clear that *Micronecta decorata* is an Oriental element ranging from northern Thailand to Borneo, Java and Sumatra (Lundblad 1933). *Micronecta ludibunda* is also widespread, ranging from India and Sri Lanka to New Guinea and the Solomon Islands (Tinerella 2008). As its closest relative, *Micronecta albifrons* (Motschulsky, 1863), known from India and Sri Lanka (Wróblewski 1968), is also considered a species of Oriental origin which has spread eastward. The four “lowland species” occurring in Borneo belong to the common Oriental elements.

Choi (1996) has pointed out that “the sedimentary basin of Mt. Kinabalu was sinuated between three crustal or tectonic plates - The South China Sea Plate to the north, the Sulu Sea Plate to the east, and the main Eurasian Plate to the west”. The uplifting of the Crocker-Trus Madi area began 40 million years ago with its collision with these other plates. The movement slowed down about 10 million years ago, although Mt. Kinabalu is said to be still pushing up at a rate of 5 mm per year, the Crocker-Trus Madi area has been pushed up into mountain ranges. According to radiometric age determinations, Mt. Kinabalu is somewhat younger, with the cooling of its magma taking place 10–4 million years ago. *Micronecta lakimi* and *Micronecta liewi* are both from the Crocker Range and not closely related to the other species of *Micronecta* collected so far in Borneo. It is, therefore, possible that the origin of these two newly described species coincided with the rising of the Crocker Range.

In view of the endemism of various organisms in Mt. Kinabalu (Wong and Phillipps 1996), these two newly described species might also be endemic to this area. However, this point of view needs to be proved by further explorations in Sabah and Borneo, notably the confirmation whether *Micronecta lakimi* and *Micronecta liewi* are endemic in the area of Crocker Range.

## Supplementary Material

XML Treatment for Micronecta

XML Treatment for Dichaetonecta

XML Treatment for Micronecta (Dichaetonecta) decorata

XML Treatment for Micronecta (Dichaetonecta) ludibunda

XML Treatment for Micronecta

XML Treatment for Micronecta (Micronecta) lumutensis

XML Treatment for Micronecta (Micronecta) liewi

XML Treatment for Micronecta (Micronecta) lakimi

XML Treatment for Micronecta (Micronecta) skutalis

XML Treatment for Sigmonecta

XML Treatment for Micronecta (Sigmonecta) kymatista

XML Treatment for Micronecta (Sigmonecta) quadristrigata
